# Peripheral Blood Stem Cell Mobilization in Healthy Donors by Granulocyte Colony-Stimulating Factor Causes Preferential Mobilization of Lymphocyte Subsets

**DOI:** 10.3389/fimmu.2018.00845

**Published:** 2018-05-02

**Authors:** Guro Kristin Melve, Elisabeth Ersvaer, Geir Egil Eide, Einar K. Kristoffersen, Øystein Bruserud

**Affiliations:** ^1^Department of Immunology and Transfusion Medicine, Haukeland University Hospital, Bergen, Norway; ^2^Department of Clinical Science, University of Bergen, Bergen, Norway; ^3^Department of Biomedical Laboratory Sciences, Western Norway University of Applied Sciences, Bergen, Norway; ^4^Centre for Clinical Research, Haukeland University Hospital, Bergen, Norway; ^5^Department of Global Public Health and Primary Care, University of Bergen, Bergen, Norway; ^6^Division for Hematology, Department of Medicine, Haukeland University Hospital, Bergen, Norway

**Keywords:** apheresis, graft versus host disease, granulocyte colony-stimulating factor, hematopoietic stem cell mobilization, hematopoietic stem cell transplantation, immune reconstitution, living donors, peripheral blood stem cells

## Abstract

**Background:**

Allogeneic hematopoietic stem cell transplantation is associated with a high risk of immune-mediated post-transplant complications. Graft depletion of immunocompetent cell subsets is regarded as a possible strategy to reduce this risk without reducing antileukemic immune reactivity.

**Study design and methods:**

We investigated the effect of hematopoietic stem cell mobilization with granulocyte colony-stimulating factor (G-CSF) on peripheral blood and stem cell graft levels of various T, B, and NK cell subsets in healthy donors. The results from flow cytometric cell quantification were examined by bioinformatics analyses.

**Results:**

The G-CSF-induced mobilization of lymphocytes was a non-random process with preferential mobilization of naïve CD4^+^ and CD8^+^ T cells together with T cell receptor αβ^+^ T cells, naïve T regulatory cells, type 1 T regulatory cells, mature and memory B cells, and cytokine-producing NK cells. Analysis of circulating lymphoid cell capacity to release various cytokines (IFNγ, IL10, TGFβ, IL4, IL9, IL17, and IL22) showed preferential mobilization of IL10 releasing CD4^+^ T cells and CD3^−^19^−^ cells. During G-CSF treatment, the healthy donors formed two subsets with generally strong and weaker mobilization of immunocompetent cells, respectively; hence the donors differed in their G-CSF responsiveness with regard to mobilization of immunocompetent cells. The different responsiveness was not reflected in the graft levels of various immunocompetent cell subsets. Furthermore, differences in donor G-CSF responsiveness were associated with time until platelet engraftment. Finally, strong G-CSF-induced mobilization of various T cell subsets seemed to increase the risk of recipient acute graft versus host disease, and this was independent of the graft T cell levels.

**Conclusion:**

Healthy donors differ in their G-CSF responsiveness and preferential mobilization of immunocompetent cells. This difference seems to influence post-transplant recipient outcomes.

## Introduction

Allogeneic hematopoietic stem cell transplantation is increasingly used in the treatment of several diseases, especially hematological malignancies and disorders characterized by severe bone marrow failure (1–4). The treatment is associated with a risk of early death mainly due to treatment toxicity, severe early immunological complications [i.e., acute graft versus host disease (aGVHD)], and a risk of long-term morbidity mainly caused by chronic GVHD (5). Various strategies of graft manipulation have been tried to reduce the frequencies of these immunological complications, including CD34 enrichment by positive or negative selection, general T cell depletion, depletion of T cell subsets, or combined B/T cell depletion (5). The early studies showed that general T cell depletion was associated with a reduced risk of severe GVHD but an increased risk of leukemia relapse and graft failure (5), whereas more recent studies based on depletion of immunocompetent cell subsets are more promising (6–10). However, the effects of depleting subsets of immunocompetent cells from the graft will probably be influenced by the frequencies of various remaining subsets of immunocompetent cells.

Treatment with granulocyte colony-stimulating factor (G-CSF) is commonly used for mobilization of peripheral blood stem cells in healthy donors (11, 12). G-CSF has several immunomodulatory effects, and for a detailed discussion and additional references we refer to a recent review (13). First, among the important effects on T cells are G-CSF-induced preferential mobilization of naïve T cells, decreased expression of T cell activation markers as well as adhesion molecules and chemokine receptors, and finally Th2 polarization with reduced production of Th1 cytokines. The levels of regulatory T cells are increased. Second, effects on NK cells and NK cell subsets are less well characterized, but there seems to be a decreased release of pro-inflammatory cytokines. Third, the differentiation status of monocytes is altered with reduced production of pro-inflammatory cytokines and increased release of immunosuppressive IL-10. These effects seem to favor an immunosuppressive effect of G-CSF administration to healthy stem cell donors, but it should be emphasized that the question of donor heterogeneity has not been investigated in detail previously.

The aim of this study was to characterize more in detail the effects of G-CSF on the mobilization of various subsets of immunocompetent cells and to have a focus on donor heterogeneity and differences in donor response to G-CSF. Hereafter, we will use the term “G-CSF responsiveness” to express the heterogeneous changes in donor peripheral blood levels of various lymphoid cell subsets during G-CSF treatment. We have characterized in detail the peripheral blood levels of various T, B, and NK cell subsets after G-CSF stem cell mobilization for an unselected group of healthy stem cell donors. Our results showed that G-CSF treatment of healthy donors caused a preferential mobilization of immunocompetent cell subsets, donors could be classified as either strong or weak mobilizers of immunocompetent cells, and this difference in G-CSF responsiveness seemed to affect the post-transplant recipient outcomes.

## Materials and Methods

### Stem Cell Donors and Allotransplant Recipients

The following participants were included: (i) 22 consecutive healthy HLA-matched related allogeneic stem cell donors, 14 males and 8 females, median age 52.5 years (25–73) and (ii) 13 male and 7 female allogeneic stem cell recipients with hematological diseases, median age 47 years (35–69). 11 patients were diagnosed with acute myeloid leukemia (AML), 4 with aplastic anemia, 2 with chronic myeloid leukemia, 2 with myelofibrosis, and 1 with chronic lymphatic leukemia. A more detailed characterization of the allotransplant recipients is given in Table S1 in Supplementary Material. The patients represent all allotransplanted patients from a defined area in Norway (the Western, Middle, and Northern Regions) during a defined time period and receiving stem cell grafts from matched family donors; i.e., this study should be regarded as a population-based study.

### Stem Cell Mobilization and Apheresis

The donors received stem cell mobilization with the human non-glycosylated G-CSF analog Filgrastim (Neupogen; Amgen, Thousand Oaks, CA, USA) or Tevagrastim (biosimilar Filgrastim; Petah Tiqwa, Israel). The donors received a median dose of 5.4 μg/kg (range 4.1–6.7 μg/kg) twice daily. Stem cell harvest was performed when the peripheral blood stem cell count exceeded 15–20 × 10^3^/mL after 4 or 5 days with either large volume apheresis using Cobe Spectra cell separator version 7 (Terumo BCT Inc., Lakewood, CO, USA; 8 donors) or automated large volume MNC procedure using Spectra Optia cell separator version 9 (Terumo BCT Inc., Lakewood, CO, USA; 14 donors).

### Allogeneic Stem Cell Transplantation

At the time of transplantation 11 patients were in their first complete hematological remission, 2 patients were in their second complete remission and 7 patients had detectable disease (Table S1 in Supplementary Material). 10 patients received myeloablative conditioning with intravenous busulfan plus cyclophosphamide (i) and 10 patients received reduced intensity conditioning with intravenous fludarabine plus busulfan (ii). After transplantation, all patients received GVHD prophylaxis with cyclosporine A plus methotrexate.

### Sample Collection and Preparation

#### Blood and Allograft Sampling

Venous blood samples from the allogeneic stem cell donors were collected (I) prior to G-CSF treatment at the time of the pre-transplant evaluation (median 20.5 days before apheresis). Blood samples were also drawn (II) in the morning immediately before apheresis, (III) immediately after apheresis, and (IV) approximately 24 h after start of apheresis. Samples for cell preparation were collected into ACD-A tubes with sodium citrate and acid-citrate-dextrose solution A as anticoagulants (Greiner Bio-One GmbH, Kremsmünster, Austria). Samples from stem cell allografts were transferred to plastic tubes without additives.

#### Cryopreservation of PBMC Samples

After isolation by density-gradient centrifugation (Lymphoprep, AXIS-SHIELD PoC AS, Oslo, Norway; specific density: 1.077 g/mL), PBMCs were dissolved in RPMI 1640 medium supplemented with 2 mmol/L l-glutamine, penicillin 100 IE/mL, streptomycin 0.1 mg/mL (Sigma-Aldrich, St. Louis, MO, USA), and 20% inactivated fetal bovine serum (Biowest, Nuaillé, France). Dimethyl sulfoxide 10% (Sigma-Aldrich, St. Louis, MO, USA) was used as a cryoprotectant, and the vials were stored in liquid nitrogen at −150°C after gradual cooling to −80°C in a Mr. Frosty Freezing Container (Thermo Fisher Scientific, Waltham, MA, USA).

### Preparation and Flow Cytometry Analyses of Peripheral Blood Mononuclear Cells

(a) All PBMC samples were thawed in a 37°C water bath, dissolved in supplemented RPMI 1640 medium and incubated for 1 h (37°C, a humidified atmosphere of 5% CO_2_) before incubation with Near-IR fluorescent reactive dye (LIVE/DEAD Fixable Dead Cell Stain Kit, Molecular Probes, Eugene, OR, USA) for 30 min. After washing in phosphate-buffered saline with 1% bovine serum albumin fraction V (Roche Diagnostics GmbH, Mannheim, Germany), the cells were incubated for 20 min with the following mouse anti-human monoclonal antibodies: CD3-PE-Cy7 (SK7), CD3-V450 (UCHT1), CD4-PerCP-Cy5.5 (RPA-T4), CD8-V500 (RPA-T8), CD16-Ax647 (3G8), CD19-PerCP-Cy5.5 (SJ25C1), CD24-PE-Cy7 (ML5), CD25-PE (M-A251), CD26-FITC (M-A261), CD27-FITC (M-T271), CD45-RA-V450 (HI100), CD56-PE (B159), CD45-RO-PE (UCHL), CD197/CCR7-FITC (150503), CD197/CCR7-Ax647 (150503), T cell receptor (TCR)αβ-BV510 (T10B91.A), TCRγδ-PE-Cy7 (11F2), iNKT(Vα24)-FITC (6b11) (all from Becton Dickinson Biosciences; BD Pharmingen, San Diego, CA, USA), CD49b-FITC (AK7; BioLegend, San Diego, CA, USA), LAG-3-PE (FAB2319P; R&D systems, Minneapolis, MN, USA), and mouse anti-human CD38-PB (HIT2; EXBIO, Prague, the Czech republic).
(b) Samples for quantification of Treg cells were thawed and surface stained as described earlier before fixation and permeabilization using eBioscience Staining Buffer Set (00-5523) as recommended by the manufacturer (eBioscience, San Diego, CA, USA). Intracellular staining was performed by incubating the cells for 30 min with mouse anti-human FoxP3-Ax647 (236A/E7; Becton Dickinson Biosciences).
(c) Samples for intracellular cytokine analyses were thawed as described in (a). The cell concentration was adjusted to 10^6^ cells/mL before stimulation for 5 h with leukocyte activation cocktail with BD GolgiPlug 2 μL/mL (PMA, Ionomycin and Brefeldin A) from Becton Dickinson Biosciences at 37°C in a humified atmosphere of 5% CO_2_. The cells were surface stained as described in (a) before fixation and permeabilization as described in (b) and finally incubated for 30 min with the following mouse anti-human monoclonal antibodies: IL4-Ax488 (8D4-8), IL9-Ax647 (MH9A3), IL10-APC (JES3-19F), IL17-A Ax488 (N49-653), IFNγ-V450 (B27), TGFβ (LAP)-PE (TW4-2F8) (all from Becton Dickinson Biosciences), and mouse anti-human monoclonal IL22-PE (142928) from R&D Systems (Abingdon, UK).

Flow cytometry analysis was performed using a FACS Canto II flow cytometer (Immunocytometry Systems; Becton Dickinson Biosciences, San Jose, CA, USA). Acquisition of 30,000 CD3^+^ T cells or 10,000 CD19^+^ B cells per sample was endeavored, and cytometer performance was monitored daily with Cytometer Setup and Tracking Beads (Becton Dickinson Biosciences). The data were analyzed with FlowJo software version 10.2 (FlowJo LLC, Ashland, OR, USA). The detailed gating strategy is shown in Figure S1 in Supplementary Material, and the main lymphoid cell subsets identified are presented in Table S2 in Supplementary Material together with detailed description of monoclonal antibodies. The identification of various cell subsets are also shown in Tables 1 and 2.

**Table 1:** Effect of granulocyte colony-stimulating factor (G-CSF) on peripheral blood and graft concentrations of various leukocyte subsets (*n* = 22) presented as median levels (×10^9^/L) with variation ranges in parentheses.

Leukocyte subset	Prior to G-CSF	During G-CSF	*p*	Stem cell graft	*R*/*p*
Neutrophils	3.4 (2.4–11.0)	36.8 (21.0–65.5)	<0.00005	100.6 (29.6–234.0)	0.182/0.193
Monocytes	0.5 (0.2–0.7)	1.9 (0.9–3.9)	<0.00005	35.1 (5.5–75.6)	0.062/0.659
Lymphocytes	1.7 (0.9–2.8)	3.9 (2.4–6.5)	<0.00005	78.1 (42.2–182.6)	0.195/0.170
T cells	1.25 (0.60–2.26)	2.92 (1.29–4.17)	<0.00005	53.92 (23.72–145.71)	0.316/0.052
B cells	0.15 (0.03–0.33)	0.50 (0.21–1.77)	<0.00005	13.50 (3.12–26.46)	0.357/0.033*
NK cells	0.22 (0.05–0.50)	0.25 (0.07–0.68)	NS	4.46 (1.74–14.47)	0.421/0.009**

*The Wilcoxon’s test for paired samples was used for comparison of pre-treatment and G-CSF-treated concentrations (fourth column). The correlations between pre-apheresis and graft concentrations were analyzed with Kendall’s tau-b test and the correlation coefficients (*R*) and corresponding *p*-values are presented in the rightmost column*.

*p < 0.05 and **p < 0.01.

**Table 2:** Effect of granulocyte colony-stimulating factor (G-CSF) on peripheral blood and graft concentrations of T cell subsets (*n* = 22) presented as median levels (×10^9^/L) with variation ranges in parentheses.

T cell subsets	Prior to G-CSF	During G-CSF	*p*	Stem cell graft	*R*/*p*
T helper cells (T_H_) (CD4^+^)	0.83 (0.39–1.37)	2.11 (0.92–3.47)	0.00004	41.10 (17.85–107.76)	0.337/0.038*
Cytotoxic T cells (T_C_) (CD8^+^)	0.29 (0.09–0.79)	0.58 (0.14–1.08)	0.0003	10.85 (3.37–33.15)	0.274/0.092
Naïve T_H_ (CD4^+^45RA^+^CCR7^+^)	0.45 (0.13–0.95)	1.21 (0.34–2.05)	0.00004	21.82 (7.30–60.24)	0.474/0.004**
Central memory cells (T_CM_) (CD4^+^45RA^−^CCR7^+^)	0.20 (0.09–0.39)	0.37 (0.13–0.87)	0.00007	7.38 (2.79–24.35)	0.442/0.006**
Effector memory cells (T_EM_) (CD4^+^45RA^−^CCR7^−^)	0.14 (0.06–0.28)	0.29 (0.08–0.72)	0.00004	5.44 (4.01–13.36)	0.326/0.044*
Terminally differentiated (T_TD_) (CD4^+^45RA^+^CCR7^−^)	0.05 (0.02–0.18)	0.11 (0.05–0.38)	0.00008	3.09 (1.12–8.05)	0.463/0.004**
(CD4^+^45RO^+^CD26^++^)	0.02 (0.01–0.07)	0.05 (0.02–0.20)	0.00004	0.82 (0.31–3.25)	0.474/0.004**
Naïve T_C_ (CD8^+^45RA^+^CCR7^+^)	0.13 (0.04–0.36)	0.24 (0.06–0.66)	0.0002	5.77 (1.63–12.46)	0.316/0.052
Central memory (CD8^+^45RA^−^CCR7^+^)	0.023 (0.003–0.080)	0.030 (0.007–0.137)	0.004	0.67 (0.08–3.03)	0.567/0.001**
Effector memory (CD8^+^45RA^−^CCR7^−^)	0.03 (0.01–0.10)	0.06 (0.01–0.17)	0.0002	1.05 (0.52–3.85)	0.442/0.006**
Effector (TEMRA) (CD8^+^45RA^+^CCR7^−^)	0.08 (0.02–0.41)	0.12 (0.02–0.36)	0.036	2.93 (0.88–14.35)	0.537/0.001**
(CD8^+^45RO^+^CD26^++^)	0.011 (0.001–0.088)	0.016 (0.002–0.101)	NS	0.24 (0.02–1.83)	0.637/0.0001***
*α*β T cells (CD3^+^T cell receptor (TCR)*α*β^+^)	1.18 (0.58–2.14)	2.76 (1.21–4.04)	0.00005	52.60 (20.63–140.47)	0.316/0.052
γδ T cells (CD3^+^4^−^8^−^TCRγδ^+^)	0.048 (0.004–0.118)	0.046 (0.009–0.178)	0.017	1.15 (0.30–4.20)	0.484/0.003**
Naïve T regulatory cells (CD4^+^25^+^45RA^+^FOXP3^+^)	0.010 (0.003–0.042)	0.019 (0.007–0.124)	0.00008	0.457 (0.165–1.817)	0.453/0.005**
Effector T regulatory cells (CD4^+^25^+^45RA^−^FOXP3^+^)	0.030 (0.016–0.068)	0.071 (0.027–0.178)	0.00004	1.268 (0.541–4.207)	0.453/0.005**
Type 1 regulatory (Tr1) (CD4^+^45RA^−^49b^+^LAG3^+^)	0.006 (0.002–0.018)	0.011 (0.004–0.064)	0.003	0.217 (<0.001–0.920)	0.211/0.194

*The Wilcoxon’s test for paired samples was used for comparison of pre-treatment and G-CSF-treated concentrations (fourth column). The correlations between pre-apheresis and graft concentrations were analyzed with Kendall’s tau-b test and the correlation coefficients (*R*) and corresponding *p*-values are presented in the rightmost column*.

*p < 0.05, **p < 0.01, and ***p < 0.001.

White blood differential counts were performed at Laboratory of Clinical Biochemistry, Haukeland University Hospital, Bergen, Norway by multi-angle polarized scatter separation optical flow cytometry using the Cell-Dyn Sapphire analyzer (Abbot Diagnostics, Santa Clara, CA, USA).

### Statistical and Bioinformatics Analyses

Descriptive statistics are given as median and range for non-normally distributed variables. The Wilcoxon’s test for paired samples was used for analyses of paired observations, and the independent-samples Mann–Whitney *U* test and the Chi Square test for comparison of unpaired groups. Correlations between continuous variables are given as the Kendall’s tau-b coefficient with corresponding *p*-value. J-Express (MolMine AS, Bergen, Norway) was applied for bioinformatics analyses (14). Time to reconstitution was analyzed with the Kaplan–Meier survival method with the log-rank test, Cox regression with backward selection, and competing risks analysis. All statistical analyses were performed in the standard computer software package IBM SPSS Statistics 22 (IBM Corporate, New York, NY, USA) except for the competing risks analyses that were done using Stata (StataCorp, Lakeway Drive College Station, Texas, USA).

## Results

### G-CSF Treatment of Healthy Stem Cell Donors Increased Peripheral Blood Levels Especially of Neutrophils but Also Monocytes and Total Lymphocytes

**Figure 1:**
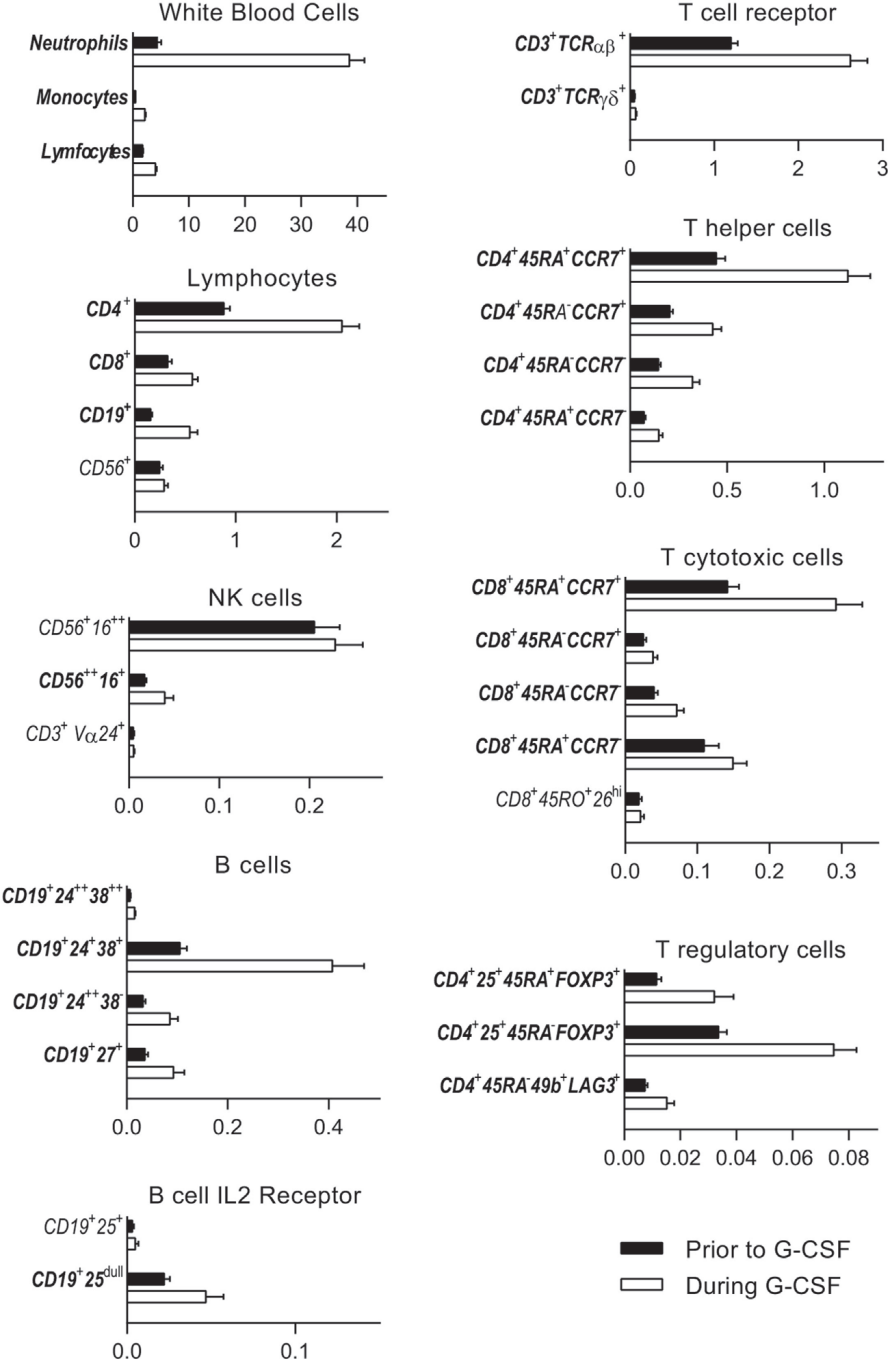
Untreated healthy donor peripheral blood immune cell concentrations (black columns) are compared to levels after granulocyte colony-stimulating factor (G-CSF) treatment (white columns). Subsets with significantly changed concentrations during G-CSF treatment are indicated with bold fonts. The peripheral blood concentrations (×10^9^/L) are given on the *x*-axes. The following subpopulations are presented: neutrophils, monocytes, and lymphocytes (CD4^+^ T helper cells, CD8^+^ T cytotoxic cells, CD19^+^ B cells, and CD56^+^ NK cells), NK cells [CD56^+^16^++^ cytolytic NK cells, CD56^++^ 16^+^ cytokine producing NK cells, and CD3^+^Vα24^+^ invariant NKT (iNKT) cells], B cells [CD19^+^24^++^38^++^ transitional B cells, CD19^+^24^+^38^+^ mature B cells, CD19^+^24^++^38^−^, and CD19^+^27^+^ memory B cells and B cell IL-2 receptor (CD25) expression], CD3^+^ T cell expression of T cell receptor *α*β and γδ, CD4^+^ and CD8^+^ memory subsets (naïve CD45RA^+^ CCR7^+^, central memory CD45RA^−^CCR7^+^, effector memory CD45RA^−^CCR7^−^, and terminally differentiated CD45RA^+^CCR7^−^), and T regulatory cells [naïve CD4^+^25^+^45RA^+^FOXP3^+^ T regulatory cells, effector CD4^+^25^+^45RA^−^FOXP3^+^ T regulatory cells, and CD4^+^45RA^−^49b^+^LAG3^+^ type 1 T regulatory cells (Tr1)]. All immune subsets with corresponding immunophenotypes and concentrations before and after G-CSF treatment are also listed in Table 2 and in Table S4 in Supplementary Material. The percentages from all flow cytometry analyses are presented in Table S4 in Supplementary Material.

**Figure 2:**
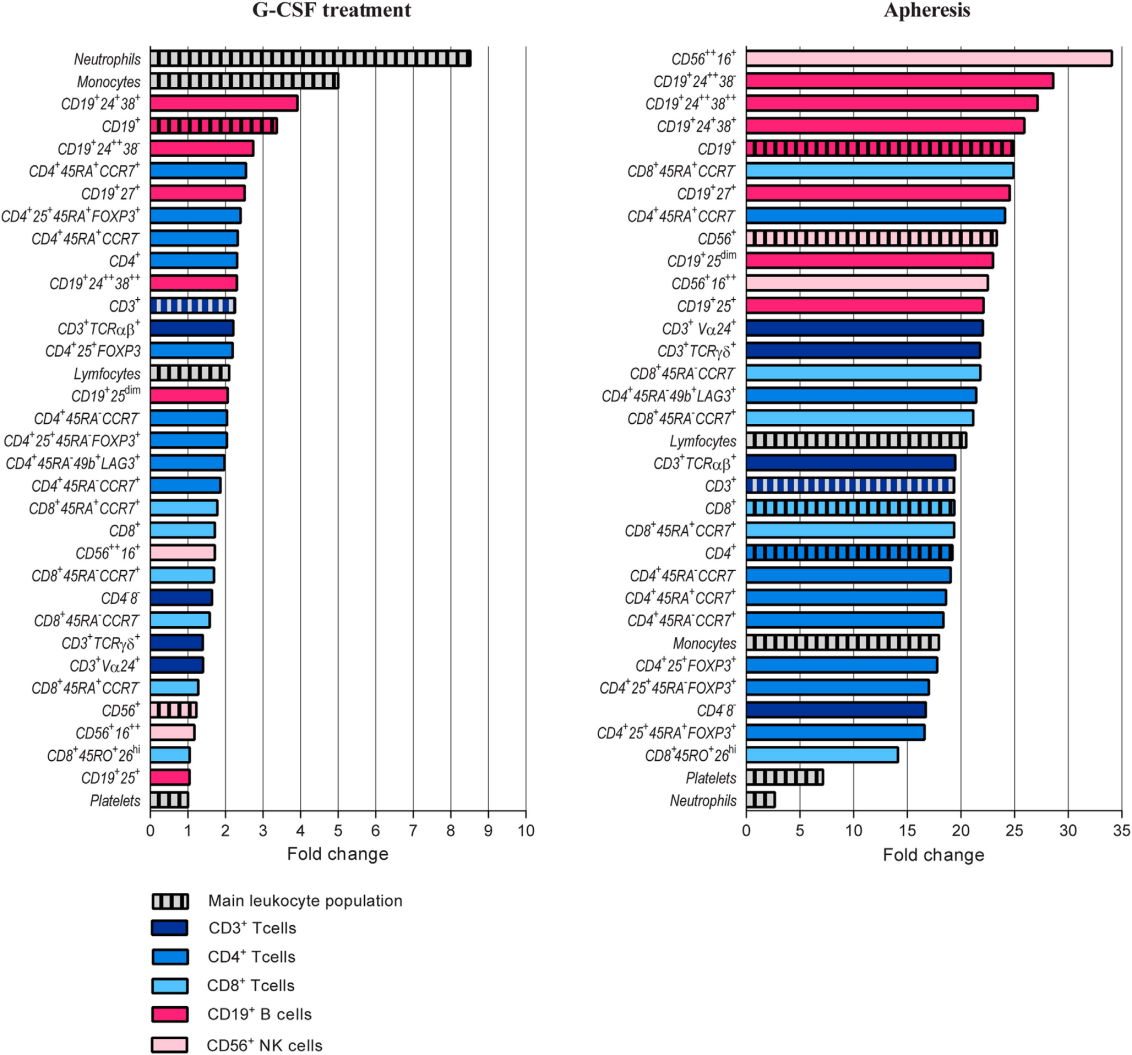
Comparison of granulocyte colony-stimulating factor (G-CSF) induced peripheral blood increments of different immune cell populations and blood platelets. T, B, and NK cell subsets are presented with different colors. The peripheral blood concentration of each subset was calculated before and after G-CSF treatment and relative change calculated. Please see Table 2 and Table S2 in Supplementary Material for classification of all immunophenotypes presented.

Granulocyte colony-stimulating factor treatment induced a five- to tenfold increase in the total peripheral blood leukocyte counts from a median level of 6.0 × 10^9^/L (range 4.4–13.4 × 10^9^/L) to 44.9 × 10^9^/L (range 26.0–71.2 × 10^9^/L). The absolute levels of virtually all leukocyte subpopulations increased (see below, Table 1; Figure 1). The increase in the proportion of neutrophils corresponded to a median fold change of 8.6, whereas the median fold change for monocytes was 5.0 and for total lymphocytes 2.1 (Figure 2; Table S3 in Supplementary Material).

### G-CSF Treatment Resulted in an Increased B Cell Fraction and Decreased NK Cell Fraction Whereas the T Cell Fraction Was Not Altered

As can be seen from Table 1 and Figures 1 and 2, there was a twofold rise in median peripheral blood T cell concentration and a threefold increase in median B cell concentration during G-CSF administration. However, the median NK cell concentration was not significantly affected by G-CSF. Consequently, there was a significant decrease in the NK cell percentage among lymphoid cells during G-CSF treatment from median 11.7 to 6.4% (*p* = 0.00006) and an increase in lymphocyte B cell percentage from median 8.4 to 10.8% (*p* = 0.0001). The change in T cell percentage from a median value of 73.3 to 69.4% was not statistically significant (Table S3 in Supplementary Material; Figure 1).

### G-CSF Increased the CD4/CD8 Ratio and the Proportion of Naïve T Regulatory Cells but Reduced the Fraction of Cytotoxic Terminally Differentiated Effector T Cells and TCRγδ^+^ T Cells

There was a significant increase in the fraction of CD4^+^ T helper cells (T_H_) in peripheral blood and an equivalent decrease in CD8^+^ T cytotoxic cells (T_C_) during G-CSF therapy (Table 2; Figure 1). The median CD4/CD8 ratio thereby increased from 2.6 (range 1.1–7.3) to 2.9 (range 1.3–7.4, *p* = 0.001) during treatment. The increased fraction of CD4^+^ cells was mainly due to an increased mobilization of naïve CD4^+^ T cells (T_N_) with a significantly reduced fraction of central memory cells.

Cytotoxic CD8^+^ T cells can be divided into at least four subsets (15, 16). G-CSF caused a preferential mobilization of naïve CD8^+^ cytotoxic T cells, and we now observed significantly reduced fractions of terminally differentiated cytotoxic CD45RA^+^ effector cell (TEMRA) and cytotoxic CD45RA^−^RO^+^ CD26^hi^ cells with unchanged central and effector memory T_C_ levels (Table 2; Figure 1; Table S4 in Supplementary Material).

Granulocyte colony-stimulating factor therapy preferentially increased the levels of circulating TCR*α*β^+^ T cells, leading to significantly reduced proportion of TCRγδ^+^ T cells. Finally, especially the levels of circulating naïve but also effector T regulatory cells and type 1 T regulatory cells (Tr1) increased during G-CSF therapy (Table 2; Figures 1 and 2).

Taken together, these observations demonstrate that G-CSF-induced T cell mobilization is not a random process with a similar effect on all T cell subsets, but rather a more selective process with preferential mobilization of naïve CD4^+^ and CD8^+^ T cells together with TCR*α*β^+^ T cells and various subsets of regulatory T cells.

### G-CSF Therapy Caused a Strong Mobilization of Mature and Memory B Cells and Decreased B Cell Expression of the IL-2 Receptor

Peripheral blood CD19^+^ B cells can be divided into the three subsets transitional, mature, and memory B cells based on the coexpression of CD24 and CD38 (17). Mature and memory B cells showed the highest fold change during G-CSF treatment of all lymphoid cell subsets examined (Figure 2). Thus, B cell mobilization is not a random process either but represents a preferential increase of certain subsets similar to the T cell mobilization. Finally, the expression of IL2 receptor on human B cells is reported to be important for their antigen presentation and T cell activation (18). During G-CSF treatment, the B cell expression of the IL2 receptor decreased, and particularly the fraction of B cells with high IL2-R expression was reduced (Figures 1 and 2; Table 3; Table S4 in Supplementary Material).

**Table 3:** Effect of granulocyte colony-stimulating factor (G-CSF) on peripheral blood and graft concentrations of B and NK cell subsets (*n* = 22) presented as median levels (×10^9^/L) with variation ranges in parentheses.

Lymphoid cell subsets	Prior to G-CSF	During G-CSF	*p*	Stem cell graft	*R*/*p*
Transitional B (CD19^+^24^++^38^++^)	0.005 (0.001–0.021)	0.013 (0.005–0.034)	0.00004	0.311 (0.087–1.045)	0.310/0.064
Mature B (CD19^+^24^+^38^+^)	0.094 (0.022–0.274)	0.352 (0.147–1.471)	0.00004	7.61 (1.52–19.09)	0.462/0.006**
Memory B (CD19^+^24^++^38^−^)	0.023 (0.002–0.097)	0.059 (0.016–0.295)	0.00004	1.898 (0.319–9.011)	0.427/0.011*
(CD19^+^27^+^)	0.027 (0.003–0.131)	0.067 (0.018–0.459)	0.00004	1.055 (0.535–13.742)	0.462/0.006**
IL-2R^+^ B (CD19^+^25^+^)	0.002 (<0.001–0.017)	0.002 (0.001–0.043)	NS	0.065 (0.012–1.028)	0.661/0.00008****
IL-2R^dull^ B (CD19^+^25^dull^)	0.017 (0.002–0.070)	0.031 (0.007–0.228)	0.0001	0.671 (0.196–6.422)	0.322/0.054
Cytolytic NK (CD56^+^16^++^)	0.191 (0.025–0.447)	0.201 (0.025–0.521)	NS	3.901 (0.882–11.986)	0.379/0.019*
Cytokine producing NK (CD56^++^16^+^)	0.018 (0.006–0.038)	0.029 (0.005–0.230)	0.001	0.619 (0.261–2.117)	0.200/0.218
Invariant NKT (CD3^+^Vα24^+.^)	0.003 (0.001–0.022)	0.003 (0.001–0.023)	NS	0.089 (0.006–2.147)	0.295/0.069

*The Wilcoxon’s test for paired samples was used for comparison of pre-treatment and G-CSF-treated concentrations (fourth column). The correlations between pre-apheresis and graft concentrations were analyzed with Kendall’s tau-b test, and the correlation coefficients (*R*) and corresponding *p*-values are presented in the rightmost column*.

*p < 0.05, **p < 0.01, ***p < 0.001, and ****p < 0.0001.

### Only Cytokine-Producing NK Cells Increased During G-CSF Therapy Whereas the Levels of Other Circulating NK Cell Subsets Were Not Altered

The peripheral blood concentrations of cytokine-producing CD56^++^CD16^+^ NK cells increased only weakly (Table 2, *p* = 0.001) during G-CSF treatment, whereas neither the level of cytolytic CD56^+^CD16^++^ NK cells nor invariant NKT (iNKT) cells showed any significant changes. Consequently, the fractions of these subsets were decreased during G-CSF therapy [i.e., immediately before stem cell apheresis (Table 3; Figure 1 and 2; Table S4 in Supplementary Material)].

### G-CSF Treatment of Healthy Donors Caused Preferential Mobilization of Certain Cytokine-Producing Lymphoid Cell Subsets

**Figure 3:**
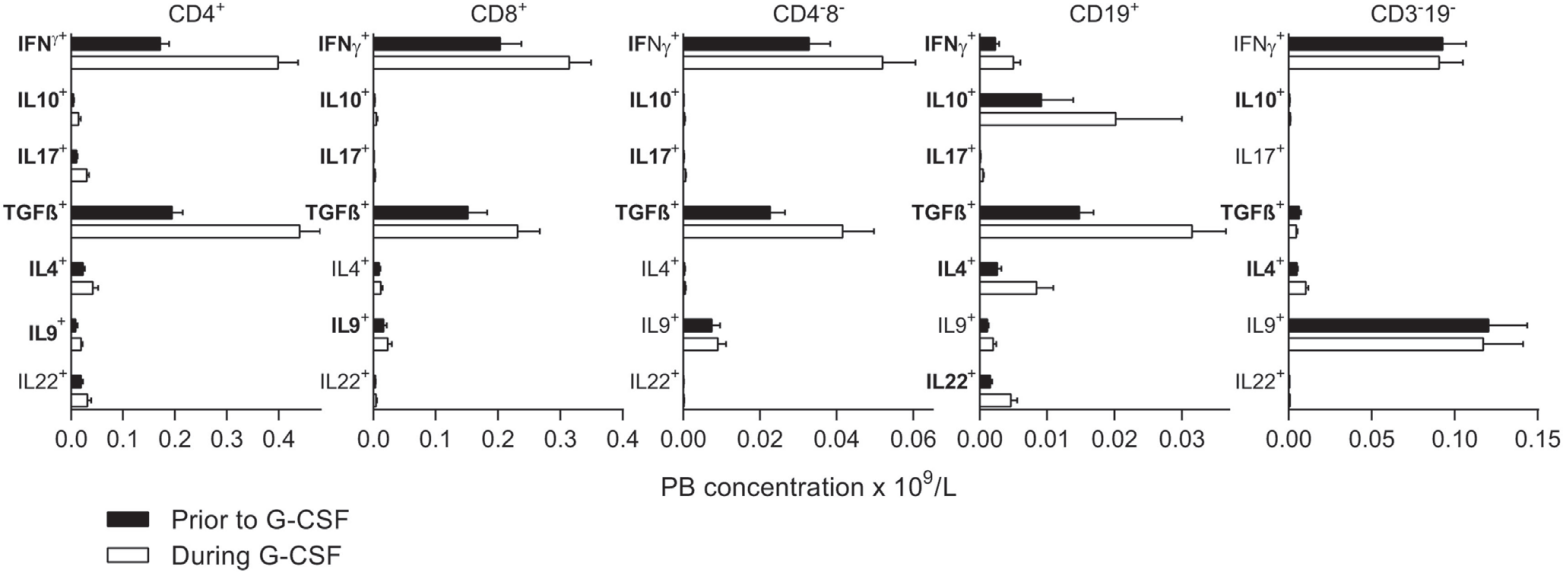
Intracellular concentrations of immunoregulatory cytokines prior to stem cell mobilization (black columns) are compared to levels after granulocyte colony-stimulating factor (G-CSF) treatment (white columns). Subsets with significantly changed concentrations during G-CSF treatment are indicated with bold fonts. The peripheral blood concentrations (×10^9^/L) are given on the *x*-axes. From left to right, results for CD4^+^ T helper cells, CD8^+^ T cytotoxic cells, CD4^−^8^−^ T cells, CD19^+^ B cells, and CD3^−^19^−^ cells are presented. The concentrations of all subsets prior to and during G-CSF treatment are also listed in Tables 4 and 5 and Table S5 in Supplementary Material. The percentages from all flow cytometry analyses are presented in Table S5 in Supplementary Material.

We investigated the intracellular levels of IFNγ, IL10, TGFβ, IL4, IL9, IL17, and IL22 in circulating main lymphoid subsets, and generally we found increased levels of cytokine-producing cells during mobilization with G-CSF (Figure 3; Tables 4 and 5). In addition to CD3^+^, CD4^+^, and CD8^+^ T cells and CD19^+^ B cells, we analyzed the cytokine production in the PB CD3^−^19^−^ and CD3^+^4^−^8^−^ populations. The CD3^−^19^−^ compartment is mainly composed of NK cells and innate lymphoid cells and the CD3^+^4^−^8^−^ subset primarily contains γδ T cells in addition to NKT cells. As shown in Table S5 in Supplementary Material, the percentage distribution of various cytokines was characteristic of each lymphoid subset, and the essential cytokine profile of each subset was conserved during G-CSF treatment. The fractions of IFNγ- and TGFβ-producing cells were high in all subsets except B cells, which showed low IFNγ and high IL10 production, and CD3^−^19^−^ cells with low TGFβ-production and high IL9 expression.

**Table 4:** Effect of stem cell mobilization with granulocyte colony-stimulating factor (G-CSF) on healthy donor T cell intracellular cytokine production (*n* = 22).

	T_H_ cells		T_C_ cells		CD3^+^4^−^8^−^ T cells	
Cytokine	Prior to/during G-CSF	*p*	Prior to/during G-CSF	*p*	Prior to/during G-CSF	*p*
IFNγ	0.157/0.374	0.00004 (↑)	0.154/0.302	0.001 (↑)	0.030/0.040	0.022 (↑)
IL10	0.0042/0.0100	0.00004 (↑)	0.0010/0.0019	0.000061 (↑)	0.0001/0.0003	0.001 (↑)
IL17	0.0091/0.0152	0.000046 (↑)	0.0008/0.0014	0.005 (↑)	0.0001/0.0004	0.007 (↑)
TGFβ	0.178/0.367	0.000061 (↑)	0.112/0.231	0.004 (↑)	0.0150/0.0297	0.024 (↑)
IL4	0.0175/0.0322	0.000367 (↑)	0.0053/0.0070	NS	0.0002/0.0002	NS
IL9	0.0048/0.0152	0.001 (↑)	0.0112/0.0107	0.011 (↓)	0.0025/0.0055	NS
IL22	0.0153/0.0211	NS	0.0033/0.0034	NS	0.0001/0.0001	NS

*From left to right, the results for T helper cells (T_H_), T cytotoxic cells (T_C_), and CD3^+^4^−^8^−^ T cells are presented. For each cytokine, the untreated concentrations (×10^9^/L) of positive cells are shown together with the concentrations during G-CSF treatment on the line below (prior to/during G-CSF), see also Figure 3. The Wilcoxon’s test for paired samples was used for comparison of pre-treatment and G-CSF-treated concentrations*.

*(↑), significant increased concentration; (↓), significant decreased concentration; NS, non-significant; *p* = *p*-value*.

**Table 5:** Effect of stem cell mobilization with granulocyte colony-stimulating factor (G-CSF) on healthy donor B and CD3^−^19^−^ cell intracellular cytokine production (*n* = 22).

	B cells		CD3^−^19^−^ cells	
Cytokine	Prior to/during G-CSF	*p*	Prior to/during G-CSF	*p*
IFNγ	0.0013/0.0036	0.002 (↑)	0.0789/0.0776	NS
IL10	0.0012/0.0041	0.001 (↑)	0.0005/0.0010	0.005 (↑)
IL17	0.0001/0.0003	0.001 (↑)	0.0001/0.0004	NS
TGFβ	0.0130/0.0249	0.000295 (↑)	0.0043/0.0033	0.022 (↓)
IL4	0.0013/0.0049	0.000069 (↑)	0.0039/0.0078	0.003 (↑)
IL9	0.0011/0.0012	NS	0.0925/0.1086	NS
IL22	0.0010/0.0030	0.000187 (↑)	0.0006/0.0006	NS

*For each cytokine, the pre-treatment concentrations (×10^9^/L) of positive cells are shown together with the concentrations after G-CSF treatment on the line below (prior to/during G-CSF), see also Figure 3. The Wilcoxon’s test for paired samples was used for comparison of pre-treatment and G-CSF-treated concentrations*.

*(↑), significant increased concentration; (↓), significant decreased concentration; NS, non-significant; *p* = *p*-value*.

Both prior to and during G-CSF administration, the highest fractions of IFNγ expressing cells were observed for T_C_. G-CSF treatment led to reduced IFNγ^+^ fractions for T_C_ cells, CD4^−^CD8^−^ T cells, and CD3^−^19^−^ cells. Furthermore, G-CSF increased the fractions of IL10 expressing T_H_, T_C_, and CD3^−^19^−^ cells. Finally, TGFβ was expressed in a large fraction of most investigated lymphoid subsets before and during mobilization, but only B cells and CD3^−^19^−^ cells showed significantly reduced TGFβ^+^ fractions during G-CSF therapy. There were generally low fractions of IL4, IL17, and IL22 expressing cells for all lymphoid subsets and these fractions remained small after G-CSF therapy, whereas for IL9 we noticed relatively large fractions within CD4^−^8^−^ T cells and especially CD3^−^19^−^ cells (Figure 3; Table S5 in Supplementary Material).

Taken together, these observations suggest that the preferential mobilization alters the overall cytokine release capacity of circulating immunocompetent cells.

### Graft Levels of Lymphoid Cell Subsets Were Increased but Reflected the Peripheral Blood Levels of Immunocompetent Cells Immediately Before Harvesting

As expected, the graft concentrations of various lymphoid cell subpopulations were generally higher than the peripheral blood levels tested immediately before apheresis, and for most lymphoid cell subsets the graft concentration represents at least a 20-fold enrichment (Figure 2; Tables 1–3). The median lymphocyte percentage corresponded to only 9.3% of circulating viable white blood cells immediately before stem cell apheresis, but increased to 36.6% in the stem cell graft. The median monocyte percentage increased to 16.2%, whereas the neutrophil percentage decreased to 42.4% (Figure 2; Table 1; Table S3 in Supplementary Material).

The fractions of B cells and monocytes among total PB leukocytes increased during G-CSF treatment, and there was an up-concentration of these two cell subsets in the grafts (corresponding to 90-fold and almost 60-fold, respectively) compared to the blood level before G-CSF administration. The T cell and especially neutrophil fractions also increased during mobilization, and the up-concentration in the graft corresponded to 45-fold and 30-fold increments compared with the pre-treatment levels. The NK cell fraction was reduced during mobilization and the graft levels of NK cells corresponded to a 20-fold increment compared to pre-treatment PB level (Figure 2; Table 1; Table S3 in Supplementary Material).

Finally, we investigated whether the PB cell subset levels immediately before apheresis showed any correlations with the corresponding graft levels (Tables 1–3). Significant correlations were detected for most cell subsets. Thus, the graft levels of immunocompetent cells in general reflected the corresponding peripheral blood levels at the time of harvesting.

### Healthy Donors Could Be Sub-Classified Based on Both the Pre-Treatment Levels and the Increase in Circulating Lymphoid Cell Subsets in Response to G-CSF Treatment

We observed a considerable variation between stem cell donors in leukocyte subset levels in peripheral blood both prior to and during G-CSF therapy. An unsupervised hierarchical clustering analysis based on untreated B, T, and NK cell concentrations identified two donor clusters (Figure S2A in Supplementary Material) characterized by significant and inverse differences in NK cell and B cell concentrations (*p* = 0.0001 and 0.0004, Mann–Whitney *U* test). Differences between donors with regard to the B/NK cell levels were maintained during G-CSF therapy (Figure S2B in Supplementary Material).

**Figure 4:**
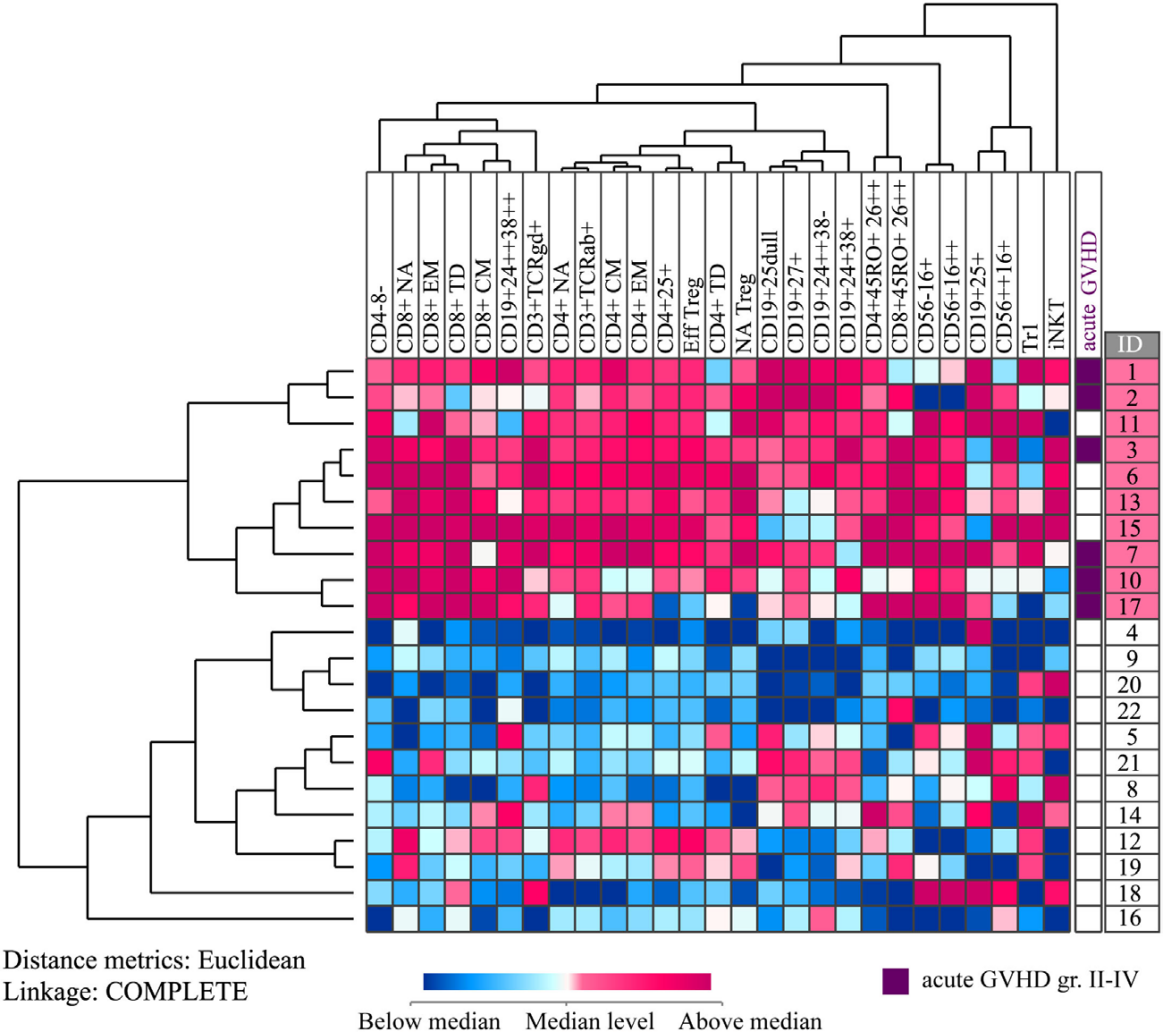
Unsupervised hierarchical cluster analyses based on healthy donor lymphocyte subset concentration changes during granulocyte colony-stimulating factor (G-CSF) treatment. All values were median normalized and log-2 transformed before performing the unsupervised hierarchical clustering analysis, and complete linkage was used as a linkage method. Euclidian distance metrics was used for distance measure. The heat map with the corresponding dendrograms is presented. Red color indicates concentration change higher than the median, whereas blue color indicates concentration change lower than the median. The vertical donor clustering into two main clusters is presented to the left of the heat map, whereas the rightmost column presents the donor identification numbers of the two clusters marked with different colors. The prevalence of acute graft versus host disease (GVHD) grades II–IV in the recipients of the donor cells is presented in a separate column to the right of the heat map. The six donors to the recipients diagnosed with this complication are marked with purple color.

We also performed unsupervised hierarchical clustering based on concentration changes in immunocompetent cells during G-CSF therapy (i.e., the ratio between pre-harvest PB concentrations and the concentrations prior to G-CSF administration for each immune cell subset), and again we identified two main donor subsets characterized by a generally strong or weak immune cell mobilizing effect of G-CSF (Figure 4). The donors in the upper cluster had significantly stronger effects of G-CSF compared to the donors in the lower cluster, and a greater increase in the peripheral blood cell concentration than in the lower cluster was seen for all lymphoid cell subsets except Tr1, iNKT cells, and CD25^+^ B cells. The most significant differences in G-CSF-induced concentration alterations were seen for TCR*α*β^+^ T cells and T cytotoxic effector memory cells (Mann–Whitney *U* test; *p* = 0.000006), T helper effector memory cells and CD3^+^4^−^8^−^ cells (*p* = 0.00002), and T helper central memory cells (*p* = 0.00004).

We investigated whether the main clusters identified in these two analyses (i.e., pre G-CSF lymphocyte concentration and G-CSF responsiveness) differed with regard to donor age, gender, ethnicity, previous diseases (especially autoimmune diseases), G-CSF dose, peripheral blood and graft CD34^+^ cell concentration, donor yield, infused dose of CD34^+^ cells per kilogram to the patients and graft content of all identified cell subsets. However, no significant differences were then observed for any of these variables when comparing the two main clusters in each of the two hierarchical clustering analyses (data not shown).

### Graft Levels of Immunocompetent Cell Subsets Did Not reflect the Corresponding Alterations in Circulating Lymphoid Cell Subsets During G-CSF Mobilization

We investigated whether the G-CSF-induced alteration in PB concentrations of various immunocompetent cell subsets (i.e., their cell concentration increments or G-CSF responsiveness) showed any correlations with the corresponding graft concentrations. However, these analyses did not show significant correlations for any of the cell subsets. Thus, the graft concentrations of immunocompetent cell subsets do not reflect the pre-apheresis donor responsiveness to G-CSF immune cell mobilization. Furthermore, we also compared the two donor subsets identified in the clustering analysis of G-CSF responsiveness (Figure 4), and these two donor subsets did not differ significantly with regard to graft concentrations or infused cell doses of CD34^+^ cells or of any immunocompetent cell subset or with regard to any of the donor characteristics mentioned above. Both these analyses suggest that the differences in donor responsiveness to G-CSF treatment (i.e., qualitative characteristics) are not reflected in the graft concentrations (i.e., quantitative characteristics) of immunocompetent cell subsets.

### G-CSF Responsiveness in Mobilization of Various Immunocompetent Cell Subsets Was Associated With Time Until Post-Transplant Hematopoietic Reconstitution

During the first day of apheresis, the median yield of CD34^+^ hematopoietic stem cells corresponded to 5.1 × 10^6^ per kg donor weight (range 0.8–22.4 × 10^6^/kg) and showed a negative correlation to donor weight (*R* = −0.481, *p* = 0.001). The stem cell products from 20 of the 22 healthy donors were transplanted to the recipients as planned, whereas the two last transplantations were canceled due to disease progression. The median total stem cell dose infused was 5.6 × 10^6^ per kg patient’s body weight (range 3.9–8.2 × 10^6^/kg). Neutrophil reconstitution with stable peripheral blood neutrophils >0.5 × 10^9^/L was achieved by 18 of the 20 recipients after a mean of 17 days (range 8–26 days). Furthermore, stable platelet reconstitution with peripheral blood levels exceeding 50 × 10^9^/L was achieved by 15 recipients after a mean of 18 days (range 12–39 days).

We investigated whether the donor subsets identified based on the pre-treatment levels of lymphocytes (Figure S2A in Supplementary Material) or the G-CSF induced concentration increase in various lymphocyte subsets (Figure 4) differed with respect to recipient neutrophil and platelet reconstitution. The recipients corresponding to donors in the upper cluster in Figure S2A in Supplementary Material (*n* = 9) had mean time to neutrophil reconstitution of 20 days (range 17–26 days), whereas the recipients corresponding to the lower cluster (*n* = 9) achieved neutrophil reconstitution after mean 16 days (range 8–18). Two patients died early before reconstitution. We did a multivariate analysis of predictors potentially influencing time to neutrophil reconstitution using Cox regression and including all 20 patients. Patients that died and patients without reconstitution were treated as censored observations. The potential predictors included patient age and gender, female to male transplantation, myeloablative versus reduced intensity conditioning, acute GVHD prophylaxis (completed methotrexate prophylaxis versus reduced methotrexate dose), infused stem cell dose, infused total leukocyte dose, ABO incompatibility, disease diagnosis, disease stage according to the EBMT index (19), and patient classification based on the donor clustering in Figure S2A in Supplementary Material. In the Cox regression of time to neutrophil reconstitution, the following four variables remained significant predictors after backward selection at significance level 0.05 in the likelihood ratio test: ABO incompatibility [HR = 11.74, 95% CI: (1.84, 75.70), *p* = 0.004], patient age [HR = 1.12, 95% CI: (0.99,1.26), *p* = 0.037], conditioning regimen [myeloablative or reduced intensity conditioning, HR = 7.48, 95% CI: (0.78,71.20), *p* = 0.045] and pre-transplant remission status [first complete remission, second complete remission or detectable disease [HR1 = 9.45:95% CI: (0.96,92.71), HR2 = 4.81; 95% CI: (0.46, 50.45), *p* = 0.050].

We also compared the hematopoietic reconstitution for the two donor clusters/subsets identified in Figure 4 (G-CSF induced concentration increase in peripheral blood cell levels). These donor/patient subsets did not differ with respect to neutrophil reconstitution. Of the 15 patients who achieved platelet counts above 50 × 10^9^/L during the first 7 weeks seven belonged to the upper donor cluster that was characterized by a generally large G-CSF induced increase in the peripheral blood levels of all immunocompetent cell subsets, and their mean time until platelet reconstitution was 21 days (range 15–39 days). Eight of the 15 patients recipients belonged to the lower donor cluster had a mean time until platelet reconstitution of 15 days (range 12–17 days). Two patients died early before reconstitution, one patient never had platelet counts below 50 × 10^9^/L (registered as missing data), and two patients showed delayed platelet reconstitution. Similar to our analysis of neutrophil reconstitution (see above), we did a multivariate analysis of factors potentially influencing platelet reconstitution, including all the variables listed above (patient age and gender, female to male transplantation, myeloablative versus reduced intensity conditioning, acute GVHD prophylaxis, infused stem cell dose, infused total leukocyte dose, ABO incompatibility, disease diagnosis, disease stage according to the EBMT index (19), and patient classification corresponding to the donor clustering presented in Figure 4). In the Cox regression of time to platelet reconstitution, the following two variables remained significant predictors: ABO incompatibility [HR = 16.0, 95% CI: (1.64,156), *p* = 0.002] and overall donor G-CSF responsiveness in terms of G-CSF-induced concentration change [see Figure 4, HR = 4.54, 95% CI: (1.25, 16.5), *p* = 0.017]. Thus, the donor G-CSF responsiveness seems to be one of the factors important for the hematopoietic reconstitution.

### Post-Transplant Outcomes Differ for Patients Receiving Allografts From Donors With Generally Strong and Weak Mobilization of Immunocompetent Cells in Response to G-CSF

After allogeneic stem cell infusion, the patients were observed until death or study closure; the median observation time was 701 days (variation range 19–1944 days). All survivors had been observed for at least 1160 days. Six recipients were diagnosed with acute GVHD grade II–IV, and all their donors belonged to the upper cluster in Figure 4 characterized by great G-CSF-induced increase, i.e., strong G-CSF responsiveness (*p* = 0.001, Pearson Chi-Square test).

**Figure 5:**
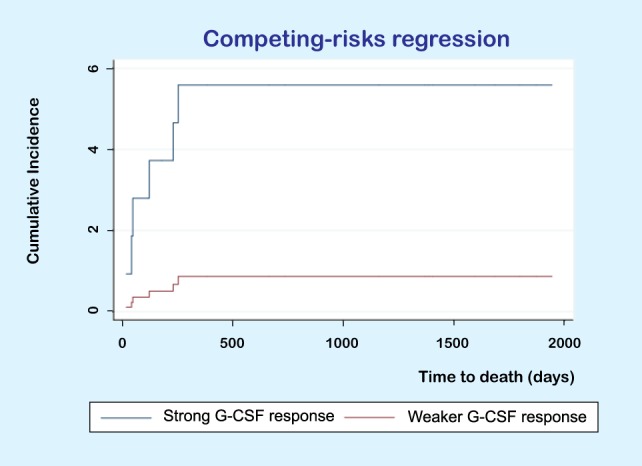
Competing risk analysis of time to death. The figure shows the results for a competing risk analysis of time to death due to toxicity (i.e., non-relapse mortality) or relapse. Patients receiving PBMC allografts from donors with a strong granulocyte colony-stimulating factor (G-CSF) response had a higher risk of non-relapse (*p* = 0.031) but donor responsiveness did not significantly influence the risk of relapse (*p* = 0.121).

We also compared the recipient mortality for the two donor subsets identified in Figure 4 (response to G-CSF) using the Kaplan–Meier method. The two recipient subsets corresponding to these two donor clusters did not differ significantly in overall survival. However, the causes of death differed between the two groups. For the donors/patients in the upper cluster, one patient died of relapse but five patients died from other causes, whereas for the patients in the lower subset five patients died from relapse and one patient died after retransplantation for graft failure. Thus, there was a different distribution of relapse and non-relapse mortality for the patients corresponding to these two donor clusters/subsets. In a competing risk analysis of time to non-relapse or relapse death, we found that patients receiving stem cell grafts from donors with strong G-CSF responsiveness had a higher risk of non-relapse death compared to recipients of grafts from donors with weaker G-CSF responsiveness (Figure 5, *p* = 0.031), but the donor G-CSF responsiveness did not have any effect on time to death due to relapse (*p* = 0.121).

## Discussion

In the present study, we describe hematopoietic stem cell mobilization in healthy donors as a heterogeneous process both with regard to differences between donors in pre-mobilization levels of circulating immunocompetent cell subsets, the general donor responsiveness to G-CSF with respect to mobilization of immune cell subsets, and differences in mobilization between various immune cell subsets (i.e., preferential mobilization). Our observations suggest that such differences may have an impact on the post-transplant outcome of the graft recipients.

We investigated an unselected group of allotransplanted patients from a defined geographic area during a defined time period and receiving peripheral blood stem cell grafts from matched family donors; this study should therefore be regarded as population-based and including well-characterized patients with a limited heterogeneity with regard to conditioning treatment, stem cell donors, graft preparation, and posttransplant handling with regard to GVHD and antibiotic prophylaxis. We would therefore emphasize that future studies have to clarify whether our results are representative also for other allotransplant recipients, i.e., patients with matched unrelated donors, other conditioning regimens, other GVHD or antibiotic prophylaxis, or other diagnoses.

The immune system represents an interactive network of a wide range of immunocompetent cell subsets. Clustering analysis is a methodological approach to identify such network-mediated interactions and correlations/covariations, and these covariations can then be a basis for identification of patient or donor subsets showing biological similarities. In the present study, clustering analyses could be used to identify distinct donor subsets based on analysis of their responsiveness to G-CSF.

The preferential G-CSF induced mobilization of several T, B, and NK cell subsets is also reflected in the graft. Graft manipulation either as *ex vivo* positive or negative selection, *in vivo* depletion of T cells by anti-thymocyte globulin or *in vivo* donor immunomodulation prior to harvesting are now considered as possible strategies for graft manipulation of healthy donors (5–10, 20–25). This study shows that donors/grafts differ in their content of various immunocompetent cell subsets, and a detailed characterization of these cells in stem cell allografts will probably be a necessary basis for optimally designed allografts. Previous studies of immunocompetent cells in G-CSF-mobilized grafts (13, 26–28) as well as more recent studies investigating associations between graft immunocompetent cells and recipient outcome have focused on selected immunocompetent cell subsets (26, 29–34), whereas we examined a wider profile of immunocompetent cells and included a focus on their G-CSF responsiveness.

Our results suggest that G-CSF therapy induces a preferential mobilization of immunocompetent cells. Relatively weak mobilizing of certain cell subsets may be important for the post-transplant clinical course of the allotransplant recipients. First, TCRγδ^+^ T cells and NK cells seem to be important for the risk of aGVHD (35–37). Second, high numbers of CD8^+^ CD45RO^+^ CD26^++^ cells in autografts are important for the risk of relapse/progression (38), whereas TEMRA is associated with a risk of cGVHD (39). Third, IL-2R-expressing B cells play a role in T cell activation and may have a role in the pathogenesis of aGVHD (18). Finally, reduced fractions of iNKT cells and preferential mobilization of naïve T_H_ may increase the risk of aGVHD (40, 41), but the preferential mobilization of CD4 cells also includes regulatory T cell subsets with immunosuppressive effects (42). Thus, the final effect of the reduced mobilization of these functionally different lymphoid subsets is difficult to predict but may represent an immunosuppressive effect. The effect of G-CSF on the cytokine release by immunocompetent cells has only been examined in a few previous studies (43–47); our present detailed characterization suggests that G-CSF therapy also alters the cytokine release profile of immunocompetent cells.

We did not find any associations between the infused dose of various immune cell subsets and the clinical outcome of the recipients, and results from previous studies of associations between cell subset dose and outcome are also conflicting (29, 30, 33, 48–50). Our present results support previous studies suggesting that the balance between different immunocompetent cell subsets is important (31, 32, 37, 51) and in addition our results suggest that the broader immunocompetent cell subset profile as well as the dose-independent responsiveness to G-CSF (i.e., the increase in the concentrations of various subsets, Figure 4) are more important than differences in single cell subset levels. Dhedin et al. previously reported that the individual donor response to G-CSF with regard to CD34^+^ stem cell mobilization was the best predictor of later aGVHD (52), but we could not confirm this. However, we also observed an association between donor responsiveness to G-CSF and aGVHD (Figure 4), i.e., a generally strong G-CSF-induced mobilization of immunocompetent cells (especially T cell subsets) in the donor was associated with increased risk of aGVHD for the recipient. The G-CSF responsiveness showed no association with the concentrations of various immunocompetent cells in the stem cell grafts, and this last observation suggests that the impact of G-CSF responsiveness is not caused simply by quantitative differences of reinfused immunocompetent cells to the transplant recipients.

The possible importance of the overall CD34^+^ stem cells dose and T cell dose for engraftment, aGVHD, and survival is still uncertain, and results from previous studies are conflicting (53–57). One possible explanation could be that the described impact of donor responsiveness to G-CSF represents an additional and dose-independent mechanism that differs between donors and thereby between recipients. Another explanation could be differences in patient inclusion, e.g., one study included only AML patients (30), whereas our study was population-based but included only patients with family donors.

We identified two main donor clusters based on the responsiveness to G-CSF (Figure 4), but at the same time the grafts from these two donor subsets did not differ with regard to the amount of CD34^+^ cells or immunocompetent cell subsets. The most likely explanation for our observed effects of donor heterogeneity on reconstitution/non-relapse mortality in the absence of quantitative differences in the number of reinfused cells is qualitative differences between the grafts. One would expect immunocompetent graft cells to exert their effects on outcome during the early post-transplant period, and several previous studies suggest that this is a critical period with regard to later complications. First, the clinical experience suggests that GVHD prophylaxis should start pre-transplant; this is true both when using prophylaxis based on anti-thymocyte globulin and cyclosporine (58). Second, post-transplant cyclophosphamide as well as methotrexate prophylaxis also start early post-transplant (58, 59). Third, the adverse effects of G-CSF treatment after allogeneic stem cell transplantation seem to depend on the biological context early after graft infusion and the use of total body irradiation in the conditioning treatment; this is supported both by clinical and experimental studies (60–62). Finally, the adverse effects of post-transplant G-CSF therapy was not seen for patients receiving G-CSF mobilized stem cell grafts, i.e., graft cells where one would expect the post-transplant effects of G-CSF to be limited because the cells had already been exposed to G-CSF before and during graft preparation. All these previous observations support our hypothesis that activation/qualitative differences between donors with regard to infused donor immunocompetent cells (i.e., their responsiveness to G-CSF) can influence the posttransplant outcome.

The immunological heterogeneity of the donors is evident both prior to and during G-CSF therapy. Platelet engraftment seems to be predicted by the intrinsic G-CSF immune cell mobilizing effect, and engraftment in the patient is influenced by both G-CSF-dependent and G-CSF-independent characteristics. The time to platelet engraftment was longer in recipients of the most G-CSF responsive donors, an apparent paradox as T cell depletion increases the risk of graft failure (63, 64). However, experience from autologous transplantation shows that T cells are less important for engraftment, when the stem cell dose is sufficient (65), and the absolute concentrations or infused doses of any immune cell subset did not differ between the G-CSF high and low responsive donor groups in this study. Furthermore, several immune cell subsets have been shown to facilitate engraftment without increasing the risk of acute GVHD through mechanisms that are not yet known (66). In line with this, intrinsic donor responsiveness to G-CSF may represent a separate mechanism that can increase the risk of recipient acute GVHD but at the same time tend to prolong time to engraftment.

Stem cell harvest by leukapheresis also contributes to the immune cell composition and activation status of the stem cell graft. Immunomodulatory effects of apheresis procedures are taken advantage of in therapeutic apheresis (67–71). Not only the mobilization but also the collection of stem cells results in a skewed distribution of different immune cell subsets that may represent a separate immunomodulatory mechanism.

In addition to detailed characterization of various lymphoid subsets, we also detected increased monocyte:lymphocyte ratio during G-CSF therapy, and stem cell mobilization with G-CSF has been shown to give preferential mobilization of CD34^+^ regulatory monocytes as well as monocytic myeloid-derived suppressor cells (34, 72–76). Monocytic and lymphoid cells are not easily separated by leukapheresis, and consequently a large fraction of monocytic cells are infused during transplantation and probably contributes to the immunomodulatory effect the stem cell graft. Several studies have demonstrated that the levels of CD34^+^ regulatory monocytes as well as monocytic myeloid-derived suppressor cells are associated with the risk of post-transplant GVHD (34, 75, 76). However, the immunosuppressive effect of monocytic cells is considered to be a double-edged sword (75), and in autologous stem cell transplantation high fractions of monocytes in the graft have been shown to have a negative effect on overall survival (77).

We observed a difference in post-transplant outcomes between the two patient clusters/subsets identified by the analysis of donor G-CSF responsiveness (Figure 4). First, for neutrophil reconstitution ABO incompatibility, patient age, conditioning regimen, and pre-transplant remission status were significant predictors after multivariate Cox regression analysis, whereas the donor differences did not have any influence. Second, for platelet reconstitution we observed an independent effect of differences in donor G-CSF responsiveness in addition to the effect of ABO incompatibility. Finally, the two clusters identified in Figure 4 showed similar early recipient mortality and no statistically significant difference in median overall survival. However, the cause of recipient death differed significantly between the two donor clusters; for the upper cluster only one out of six patients died from relapse, whereas for the lower cluster five out of six patients did so. Our competing risk regression analysis also showed an association of borderline significance between high G-CSF responsiveness and non-relapse mortality. Taken together, these observations suggest that immunological differences between donors with regard to G-CSF responsiveness are important for recipient outcome after allotransplantation. However, due to our low number of donors/recipients, we would emphasize that our observations need to be confirmed in larger clinical studies.

In conclusion, our study gives one of the most detailed characterizations of the immunomodulatory effects of stem cell mobilization and apheresis on the distribution of multiple lymphoid cell subsets available this far and shows that donor immune characteristics may be important for recipient outcome. Both G-CSF treatment and apheresis skew the distribution of various immune cell subsets and thereby influence graft composition, and both G-CSF dependent and independent immunological heterogeneity of the donors are reflected in the outcome of the patients. The results of our study indicate that the intrinsic effect of G-CSF on donor immune cell mobilization is associated with the reconstitution of platelets and the prevalence of acute GVHD after related HLA-matched stem cell transplantation. As this study includes relatively few participants, these results need to be confirmed in larger studies.

## Ethics Statement

This study was carried out in accordance with the recommendations in the guidelines drawn up by The Norwegian National Research Ethics Committee for medical and health research (NEM) with written informed consent from all subjects. All subjects gave written informed consent in accordance with the Declaration of Helsinki. The protocol was approved by the Regional Committee for Medical and Health Research Ethics of Western Norway (2011/996, 2011/1237, 2011/1241 and 2013/634).

## Author Contributions

ØB, EE, GM, and EK designed the study. GM performed the laboratory experiments trained by EE and with advice from EK. GM analyzed the flow cytometry data and performed basic statistical and bioinformatics analyses. GE performed the Cox regression and competing risk analyses. GM, ØB, EE, EK, and GE evaluated the results. GM and ØB wrote the paper, and EK, GE, and EE critically revised the manuscript.

## Conflict of Interest Statement

The authors declare that the research was conducted in the absence of any commercial or financial relationships that could be construed as a potential conflict of interest.
