# Supplementary material for: Peripheral Blood Stem Cell Mobilization in Healthy Donors by Granulocyte Colony-Stimulating Factor Causes Preferential Mobilization of Lymphocyte Subsets
Source: Front Immunol. 2018 May 2;9:845. doi: 10.3389/fimmu.2018.00845 (PMC5941969; doi:10.3389/fimmu.2018.00845)
Supplement: Supplementary file 1 [file Data_Sheet_1.zip › Legends to Supplementary Figures 1 and 2.DOCX]

Supplementary Material

Peripheral Blood Stem Cell Mobilization in Healthy Donors by Granulocyte Colony-Stimulating Factor Causes Preferential Mobilization of Lymphocyte Subsets

Guro Kristin Melve, Elisabeth Ersvaer, Geir Egil Eide, Einar K. Kristoffersen, Øystein Bruserud^*^

*** Correspondence:** Corresponding Author: Prof. Øystein Bruserud: oystein.bruserud@haukeland.no

# Legends to Supplementary Figures

## Legend to Supplementary Figure 1

*Gating strategy for lymphoid subsets.* The selection and identification of subsets is described from left to right for all panels:

Panel (A): Initial gating of all events included the lymphocyte gate followed by the selection of singlets based on FSC-H (forward scatter-Height) and FSC-W (forward scatter-With) plus SSC-A (side scatter-Areal) and SSC-H (side scatter-Height). All lymphocytes were evaluated for viability by use of Near-IR fluorescent reactive dye. Live CD3^+^ T cells were separated from live CD3^-^ lymphocytes with CD3 PECy7, except from in initial characterization of NK cell subsets (Panel (C)), CD3 V450 was then used.

Panel (B): With a CD4/CD8 four-quadrant gate on selected CD3^+^ cells four T cell subsets could be identified: (i) CD4^+^8^-^ T helper cells (ii) CD4^-^8^+^ T cytotoxic cells (i) CD4^+^8^+^ double positive T cells and CD4^-^8^-^ double negative T cells. A four-quadrant gate was also used for identification of naïve and memory T helper cells based on CD45-RA and CCR7 expression: (i) CD4^+^45^-^RA^+^CCR7^+^ naïve Th cells (ii) CD4^+^45^-^RA^-^CCR7^-^ effector memory (EM) Th cells (iii) CD4^+^45^-^RA^-^CCR7^+^ central memory (CM) Th cells (iv) CD4^+^45^-^RA^-^CCR7^-^ terminally differentiated (TD) Th cells. Identical gating strategy was used for identification of CD8^+^ naïve and memory cytotoxic T cells. A distinct CD45RO^+^CD26^hi^ subpopulation was identified in selected CD8^+^ cells, and the corresponding phenotype was also detected for CD4^+^ cells. Finally, the identification of T cell receptor (TCR) divergent ɑβ T cells and γδ T cells is shown.

Panel (C): CD56^+^16^++^ cytolytic and CD56^++^16^dim^ cytokine producing NK cells were selected from CD3^-^ lymphocytes and Vα24^+^  iNKT cells from CD3^+^ cells. T regulatory cells were selected from CD4^+^25^+^ cells and gated into CD45RA^+^ FoxP3^+^ naïve and CD45RA^-^ FoxP3^+^ effector T regulatory cells.

Panel (D): Type 1 regulatory (Tr1) cells were identified as CD4^+^45RA^-^49b^+^LAG3^+^. Cytokine expressing cells (IFNɣ, TGFβ and IL-10) are shown as fractions of CD4^+^ T cells. Corresponding subset identification was performed for CD8^+^ T cells, CD19^+^ B cells, CD3^-^19^-^ lymphocytes (i.e. mainly NK cells and innate lymphoid cells) and CD4^-^8^-^ T cells (i.e. mainly γδ T cells and NKT cells).

Panel (E): Identification of IL17, IL4, IL9 and IL22 expressing cells is like in Panel D shown for CD4^+^ T cells, but was also identified in CD8^+^ T cells, CD19^+^ B cells, CD3^-^19^-^ lymphocytes (i.e. mainly NK cells) and CD4^-^8^-^ T cells (i.e. mainly γδ T cells).

Panel (F): CD19^+^ B cells was identified and could be separated into CD24^+^38^+^ mature B cells, CD24^++^38^-^ memory B cells and CD24^++^38^++^ transitional B cells. Finally, the expression of IL2-R (CD25) and CD27 on all B-cells was evaluated; the IL2-R expressing cells were classified as CD25^+^ or CD25^dim^.

## Legend to Supplementary Figure 2

Unsupervised hierarchical cluster analyses based on untreated (A) and G-CSF treated (B) healthy donor T, B and NK cell PB concentrations. All values were median normalized and log-2 transformed before performing the unsupervised hierarchical clustering analyses and complete linkage was used as linkage method. The Pearson correlation was used for distance measure. The heat maps with corresponding dendrograms are presented. Red color indicates concentration higher than the median; whereas blue color indicates concentration lower than the median. The vertical donor clustering into two main clusters is presented to the left of the heat maps, while the rightmost columns present the donor identification numbers of the two clusters marked with different colors based on the donor clustering in (A). With only two exceptions (donor 4 and donor 13), the donors clustered identically into the upper and lower donor cluster during G-CSF (B).
