# Supplementary material for: Peripheral Blood Stem Cell Mobilization in Healthy Donors by Granulocyte Colony-Stimulating Factor Causes Preferential Mobilization of Lymphocyte Subsets
Source: Front Immunol. 2018 May 2;9:845. doi: 10.3389/fimmu.2018.00845 (PMC5941969; doi:10.3389/fimmu.2018.00845)
Supplement: Supplementary file 1 [file Data_Sheet_1.zip › Supplementary Tables 1-5.docx]

Supplementary Tables

Peripheral Blood Stem Cell Mobilization in Healthy Donors by Granulocyte Colony-Stimulating Factor Causes Preferential Mobilization of Lymphocyte Subsets

Guro Kristin Melve, Elisabeth Ersvaer, Geir Egil Eide, Einar K. Kristoffersen, Øystein Bruserud^*^

*** Correspondence:** Corresponding Author: Prof. Øystein Bruserud: oystein.bruserud@haukeland.no

# Supplementary Tables

## Supplementary Table 1: Clinical characteristicsof the 20 allogeneic stem cell recipients.

| **Identity**^1^ | **Gender (M/F)** | **Gender match**^2^ | **Age (years)** | **Diagnosis^3^** | **Complete remission**^4^ | **EBMT risk score^5^** | **HCT CI^6^** | **HLA match** | **ABO incompatibility** | **Conditioning^7^** | **Stem cell dose**^8^ | **WBC dose**^9^ | **aGVHD** | **Relapse** |
| --- | --- | --- | --- | --- | --- | --- | --- | --- | --- | --- | --- | --- | --- | --- |
| 1 | M | **-** | 57 | MDS^L^ | - | 6 | 1-2 | 10/10 | - | RIC | 8.2 | 16.4 | + | - |
| 2 | F | + | 52 | AML | CR1 | 2 | ≥3 | 10/10 | - | RIC | 5.6 | 18.8 | + | - |
| 3 | M | + | 43 | AML | CR1 | 2 | 1-2 | 10/10 | - | MAC | 5.1 | 8.7 | + | - |
| 4 | M | + | 43 | AML | CR1 | 2 | ≥3 | 10/10 | - | RIC | 8.0 | 4.7 | - | - |
| 5 | M | + | 64 | MDS^E^ | - | 3 | 0 | 09/10 | major | RIC | 5.9 | 24.9 | - | + |
| 7 | F | + | 45 | AML | - | 5 | ≥3 | 10/10 | minor | MAC | 6.3 | 24.6 | + | - |
| 8 | M | + | 69 | MDS^I^ | CR2 | 4 | 0 | 10/10 | - | RIC | 5.5 | 27.3 | - | + |
| 9 | F | + | 49 | AML | CR1 | 2 | 0 | 10/10 | major | MAC | 5.5 | 18.2 | - | - |
| 10 | F | + | 39 | MDS^I^ | - | 2 | 1-2 | 10/10 | - | MAC | 5.9 | 5.1 | + | - |
| 11 | M | - | 44 | AML | CR1 | 3 | 0 | 10/10 | - | MAC | 6.2 | 11.5 | - | + |
| 12 | M | + | 64 | CMF^E^ | - | 3 | 0 | 10/10 | major | RIC | 5.0 | 5.9 | - | - |
| 13 | F | + | 36 | ALL | CR2 | 3 | 1-2 | 10/10 | - | MAC | 5.0 | 3.8 | - | - |
| 14 | M | - | 44 | AML | CR1 | 3 | 1-2 | 10/10 | - | MAC | 5.0 | 16.1 | - | - |
| 15 | M | + | 60 | CMF^E^ | - | 4 | 1-2 | 09/10 | - | RIC | 3.9 | 16.6 | - | - |
| 16 | F | + | 35 | AML | CR1 | 1 | 0 | 10/10 | - | MAC | 5.2 | 5.4 | - | - |
| 17 | F | + | 41 | ALL | CR1 | 2 | 0 | 10/10 | - | MAC | 5.7 | 7.1 | + | - |
| 18 | M | + | 63 | AML | CR1 | 2 | ≥3 | 10/10 | - | RIC | 5.5 | 7.4 | - | + |
| 19 | M | - | 45 | AML | CR1 | 3 | 0 | 10/10 | - | MAC | 5.4 | 10.4 | - | + |
| 21 | M | + | 66 | CLL^L^ | - | 5 | 0 | 10/10 | - | RIC | 6.2 | 4.2 | - | + |
| 22 | M | + | 62 | AML | CR1 | 2 | ≥3 | 10/10 | - | RIC | 5.9 | 12.1 | - | - |

^1^For the patients ID6 and ID20 the planned transplantations were cancelled due to disease exacerbation.

^2^Male to male, female to female and male to female donations are indicated as +, female to male donation is indicated as -

^3^AML, acute myeloid leukemia; ALL, acute lymphoblastic leukemia; CLL, chronic lymphocytic leukemia; CMF, chronic myelofibrosis; MDS, myelodysplastic syndrome. For patients with other diseases than acute leukemic the disease stage from the EBMT risk score is indicated after the diagnosis (E, early; I, intermediate; L, late).

^4^ CR1, first complete remission, CR2, second complete remission.

^5^Gratwohl A, Stern M, Brand R, Apperley J, Baldomero H, de Witte T, et al. Risk score for outcome after allogeneic hematopoietic stem celtransplantation: a retrospective analysis. *Cancer* (2009) 115(20):4715-26. doi: 10.1002/cncr.24531. PubMed PMID: 19642176.

^6^Sorror ML, Maris MB, Storb R, Baron F, Sandmaier BM, Maloney DG, et al. Hematopoietic cell transplantation (HCT)-specific comorbidity index: a new tool for risk assessment before allogeneic HCT. Blood (2005) 106(8):2912-9. doi: 10.1182/blood-2005-05-2004. PubMed PMID: 15994282; PubMed Central PMCID: PMC1895304.

^7^MAC, myeloablative conditioning; RIC, reduced intensity conditioning.

^8^Stem cell dosesare given as x 10^6^/kg body weight of the recipient.

^9^ White blood cell doses are given as x 10^7^/kg body weight of the recipient.

## Supplementary Table 2:Definitions ofimmunophenotypes of the main T, B and NK cell subsets identified

| **Lymphoidcellsubsets** | **Immunophenotype** | **Monoclonalantibodies/Fluorochromes** | **Reference** | |
| --- | --- | --- | --- | --- |
| **T cells** |  |  | |  |
| Naïve T_H_ | CD4^+^45RA^+^CCR7^+^ | CD3-PE-Cy7 (SK7), CD4-PerCP-Cy5.5 (RPA-T4), CD45-RA-V450 (HI100), CD197/CCR7-Ax647 (150503) | | (1) |
| Central memory (T_CM_) | CD4^+^45RA^-^CCR7^+^ | CD3-PE-Cy7 (SK7), CD4-PerCP-Cy5.5 (RPA-T4), CD45-RA-V450 (HI100), CD197/CCR7-Ax647 (150503) | | (1) |
| Effectormemory (T_EM_) | CD4^+^45RA^-^CCR7^-^ | CD3-PE-Cy7 (SK7), CD4-PerCP-Cy5.5 (RPA-T4), CD45-RA-V450 (HI100), CD197/CCR7-Ax647 (150503) | | (1) |
| Terminallydifferentiated (T_TD_) | CD4^+^45RA^+^CCR7^-^ | CD3-PE-Cy7 (SK7), CD4-PerCP-Cy5.5 (RPA-T4), CD45-RA-V450 (HI100), CD197/CCR7-Ax647 (150503) | | (1) |
| Naïve T_c_ | CD8^+^45RA^+^CCR7^+^ | CD3-PE-Cy7 (SK7), CD8-V500 (RPA-T8), CD45-RA-V450 (HI100), CD197/CCR7-Ax647 (150503) | | (1) |
| Central memory | CD8^+^45RA^-^CCR7^+^ | CD3-PE-Cy7 (SK7), CD8-V500 (RPA-T8), CD45-RA-V450 (HI100), CD197/CCR7-Ax647 (150503) | | (1) |
| Effectormemory | CD8^+^45RA^-^CCR7^-^ | CD3-PE-Cy7 (SK7), CD8-V500 (RPA-T8), CD45-RA-V450 (HI100), CD197/CCR7-Ax647 (150503) | | (1) |
| Effector (TEMRA) | CD8^+^45RA^+^CCR7^-^ | CD3-PE-Cy7 (SK7), CD8-V500 (RPA-T8), CD45-RA-V450 (HI100), CD197/CCR7-Ax647 (150503) | | (1) |
|  | CD4^+^45RO^+^CD26^++^ | CD3-PE-Cy7 (SK7), CD4-PerCP-Cy5.5 (RPA-T4), CD45-RO-PE (UCHL), CD26-FITC (M-A261) | | (2) |
|  | CD8^+^45RO^+^CD26^++^ | CD3-PE-Cy7 (SK7), CD8-V500 (RPA-T8), CD45-RO-PE (UCHL), CD26-FITC (M-A261) | | (2) |
| ɑβ T cells | CD3^+^TCRɑβ^+^ | CD3-V450 (UCHT1),TCRαβ-BV510 (T10B91.A) | | (3) |
| γδ T cells | CD3^+^4^-^8^-^TCRɣδ^+^ | CD3-V450 (UCHT1),TCRγδ-PE-Cy7 (11F2) | | (3) |
| Naïve T regulatorycells | CD4^+^25^+^45RA^+^FOXP3^+^ | CD3-PE-Cy7 (SK7), CD4-PerCP-Cy5.5 (RPA-T4), CD25-PE (M-A251), CD45-RA-V450 (HI100), FoxP3-Ax647 (236A/E7) | | (4) |
| Effector Tregulatorycells | CD4^+^25^+^45RA^-^FOXP3^+^ | CD3-PE-Cy7 (SK7), CD4-PerCP-Cy5.5 (RPA-T4), CD25-PE (M-A251), CD45-RA-V450 (HI100), FoxP3-Ax647 (236A/E7) | | (4) |
| Tr1 | CD4^+^45RA^-^49b^+^LAG3^+^ | CD3-PE-Cy7 (SK7), CD4-PerCP-Cy5.5 (RPA-T4), CD45-RA-V450 (HI100), CD49b-FITC (AK7), LAG-3-PE (FAB2319P) | | (5) |
| **B cells** |  |  | |  |
| Transitional | CD19^+^24^++^38^++^ | CD19-PerCP-Cy5.5 (SJ25C1), CD24- PE-Cy7 (ML5), CD38-PB (HIT2) | | (6) |
| Mature | CD19^+^24^++^38**^-^** | CD19-PerCP-Cy5.5 (SJ25C1), CD24- PE-Cy7 (ML5), CD38-PB (HIT2) | | (6) |
| Memory | CD19^+^24^+^38^+^ | CD19-PerCP-Cy5.5 (SJ25C1), CD24- PE-Cy7 (ML5), CD38-PB (HIT2) | | (6) |
|  | CD19^+^27^+^ | CD19-PerCP-Cy5.5 (SJ25C1), CD27-FITC (M-T271), | | (6) |
| IL-2R^+^ | CD19^+^25^+^ | CD19-PerCP-Cy5.5 (SJ25C1), CD25-PE (M-A251) | | (7) |
| IL-2R^w^ | CD19^+^25^w^ | CD19-PerCP-Cy5.5 (SJ25C1), CD25-PE (M-A251) | | (7) |
| **NK cells** |  |  | |  |
| Cytolytic | CD56^+^16^++^ | CD3-V450 (UCHT1), CD56-PE (B159), CD16-Ax647 (3G8) | | (8) |
| Cytokineproducing | CD56^++^16^+^ | CD3-V450 (UCHT1), CD56-PE (B159), CD16-Ax647 (3G8) | | (8) |
| Invariant NKT (iNKT) | CD3^+^Vα24^+.^ | CD3-V450 (UCHT1),iNKT(Vα24)-FITC (6b11) | | (9) |

1. Matteucci E, Ghimenti M, Di Beo S, Giampietro O. Altered proportions of naive, central memory and terminally differentiated central memory subsets among CD4+ and CD8 + T cells expressing CD26 in patients with type 1 diabetes. *Journal of clinical immunology* (2011) 31(6):977-84. doi: 10.1007/s10875-011-9573-z. PubMed PMID: 21887518.

2. Hildebrandt M, Dijkstra D, Gollasch H, Daemen K, Stevanovic-Meyer M, Ludwig WD. Apheresis-related enrichment of CD26++ T lymphocytes: phenotypic characterization and correlation with unfavorable outcome in autologous hematopoietic progenitor cell transplantation. *Transfusion* (2012) 52(4):765-76. doi: 10.1111/j.1537-2995.2011.03351.x. PubMed PMID: 21950525.

3. Vantourout P, Hayday A. Six-of-the-best: unique contributions of gammadelta T cells to immunology. *Nature reviews Immunology* (2013) 13(2):88-100. doi: 10.1038/nri3384. PubMed PMID: 23348415; PubMed Central PMCID: PMC3951794.

4. Simonetta F, Bourgeois C. CD4+FOXP3+ Regulatory T-Cell Subsets in Human Immunodeficiency Virus Infection. *Frontiers in immunology* (2013) 4:215. doi: 10.3389/fimmu.2013.00215. PubMed PMID: 23908654; PubMed Central PMCID: PMC3727053.

5. Gagliani N, Magnani CF, Huber S, Gianolini ME, Pala M, Licona-Limon P, et al. Coexpression of CD49b and LAG-3 identifies human and mouse T regulatory type 1 cells. *Nature medicine* (2013) 19(6):739-46. doi: 10.1038/nm.3179. PubMed PMID: 23624599.

6. Carsetti R, Rosado MM, Wardmann H. Peripheral development of B cells in mouse and man. *Immunological reviews* (2004) 197:179-91. Epub 2004/02/14. PubMed PMID: 14962195.

7. Brisslert M, Bokarewa M, Larsson P, Wing K, Collins LV, Tarkowski A. Phenotypic and functional characterization of human CD25+ B cells. *Immunology* (2006) 117(4):548-57. Epub 2006/03/25. doi: IMM2331 [pii]

10.1111/j.1365-2567.2006.02331.x. PubMed PMID: 16556269; PubMed Central PMCID: PMC1782245.

8. Cooper MA, Fehniger TA, Turner SC, Chen KS, Ghaheri BA, Ghayur T, et al. Human natural killer cells: a unique innate immunoregulatory role for the CD56(bright) subset. *Blood* (2001) 97(10):3146-51. PubMed PMID: 11342442.

9. Berzins SP, Smyth MJ, Baxter AG. Presumed guilty: natural killer T cell defects and human disease. *Nature reviews Immunology* (2011) 11(2):131-42. doi: 10.1038/nri2904. PubMed PMID: 21267014.

**Supplementary Table 3:** The effect of G-CSF on peripheral blood and graft concentrations and percentages of various leukocyte subsets (n = 22) presented as median levels with variation ranges in parentheses. In the left part of the table the reults are presented as PB and graft concentrations, whereas in the right part the same values are presented as percentages of their parent populations acquired by flow cytometry. Neutrophils, monocytes and lymphocytes are presented as percentages of total WBC count, and T-, B- and NK cells as percentages of the total number of lymphocytes. All values are given as medians with variation ranges in parentheses. The Wilcoxon's test for paired samples was used for comparison of pre-treatment and G-CSF treated/pre-apheresis concentrations and percentages and pre-apheresis and graft concentrations and percentages.

| **Leukocytesubset** | **Prior to**  **G-CSF**  **(x 10^9^/L)** | **During**  **G-CSF**  **(x 10^9^/L)** | **P** | **Stem cellgraft**  **(x 10^9^/L)** | **P** | **Prior to**  **G-CSF**  **(%)** | **During**  **G-CSF**  **(%)** | **P** | **Stem cellgraft (%)** | **P** |
| --- | --- | --- | --- | --- | --- | --- | --- | --- | --- | --- |
| Neutrophils | 3.4  (2.4-11.0) | 36.8  (21.0-65.5) | 0.00004 | 100.6  (29.6-234.0) | 0.00005 | 60.5  (47.3-83.3) | 84.9  (77.7-92.2) | 0.00004 | 42.4  (13.8-68.8) | 0.00004 |
| Monocytes | 0.5  (0.2-0.7) | 1.9  (0.9-3.9) | 0.00004 | 35.1  (5.5-75.6) | 0.00004 | 7.1  (3.1-11.4) | 4.6  (2.3-8.3) | 0.001 | 16.2  (1.6-38.3) | 0.00006 |
| Lymphocytes | 1.7  (0.9-2.8) | 3.9  (2.4-6.5) | 0.00004 | 78.1  (42.2-182.6) | 0.00004 | 27.7  (8.3-42.1) | 9.3  (4.4-13.2) | 0.00005 | 36.6  (17.6-59.9) | 0.00004 |
| T-cells | 1.25  (0.60-2.26) | 2.92  (1.29-4.17) | 0.00004 | 53.92  (23.72-145.71) | 0.00009 | 73.3  (54.8-82.1) | 69.4  (44.1-81.9) | NS | 62.1  (45.9-85.2) | 0.001 |
| B-cells | 0.15  (0.03-0.33) | 0.50  (0.21-1.77) | 0.00004 | 13.50  (3.12-26.46) | 0.0001 | 8.4  (2.28-17.5) | 10.8  (5.9-29.9) | 0.0001 | 14.5  (6.1-36.5) | 0.003 |
| NK-cells | 0.22  (0.05-0.50) | 0.25  (0.07-0.68) | NS | 4.46  (1.74-14.47) | 0.00009 | 11.7  (3.0-30.9) | 6.4  (1.5-15.8) | 0.00006 | 5.6  (2.3-18.4) | 0.023 |

**Supplementary Table 4.**The effect of stem cell mobilization of 22 healthy donors with G-CSF on the concentrations and

percentages of T, B and NK cell subsets in peripheral blood and in the stem cell graft. In the left part of the table the results are

presented as PB and graft concentrations (x 10^9^/L), whereas in the right part the same values are presented as percentages of their

parent populations acquired by flow cytometry. All values are given as medians with variation ranges in parentheses. The Wilcoxon's

test for paired samples was used for comparison of pre-treatment and G-CSF treated/pre-apheresis concentrations and percentages.

| **Lymphoidcellsubsets** | **Immunophenotype** | **Prior to**  **G-CSF** | **During G-CSF** | **P** | **Prior to G-CSF** | **During G-CSF** | **P** | **Stem cellgraft**  **(x 10^9^/L)** | **Stem cellgraft**  **%** |
| --- | --- | --- | --- | --- | --- | --- | --- | --- | --- |
| **T cells** |  |  |  |  |  |  |  |  |  |
| T helpercells (T_H_) | CD4^+^ | 0.83  (0.39-1.37) | 2.11  (0.92-3.47) | 0.00004(↑) | 67.3  (49.1-84.8) | 74.5  (56.6-89.0) | 0.001(↑) | 41.10  (17.85-107.76) | 73.7  (61.8-89.4) |
| Cytotoxic T cells (T_c_) | CD8^+^ | 0.29  (0.09-0.79) | 0.58  (0.14-1.08) | 0.0003(↑) | 24.6  (11.6-46.3) | 20.4  (8.9-35.8) | 0.001 (↓) | 10.85  (3.37-33.15) | 22.7  (8.0-35.9) |
| Naïve T_H_ | CD4^+^45RA^+^CCR7^+^ | 0.45  (0.13-0.95) | 1.21  (0.34-2.05) | 0.00004(↑) | 48.4  (20.8-74.4) | 53.6  (21.3-74.0) | 0.0002(↑) | 21.82  (7.30-60.24) | 54.8  (35.0-73.5) |
| Central memory (T_CM_) | CD4^+^45RA^-^CCR7^+^ | 0.20  (0.09-0.39) | 0.37  (0.13-0.87) | 0.00007(↑) | 22.2  (15.0-40.7) | 20.6  (12.1-36.9) | 0.001 (↓) | 7.38  (2.79-24.35) | 19.4  (11.2-30.0) |
| Effectormemory (T_EM_) | CD4^+^45RA^-^CCR7^-^ | 0.14  (0.06-0.28) | 0.29  (0.08-0.72) | 0.00004(↑) | 17.4  (4.8-32.8) | 14.2  (6.2-36.0) | 0.015 (↓) | 5.44  (4.01-13.36) | 17.8  (7.9-26.2) |
| Terminallydifferentiated (T_TD_) | CD4^+^45RA^+^CCR7^-^ | 0.05  (0.02-0.18) | 0.11  (0.05-0.38) | 0.00008(↑) | 6.8  (3.0-22.2) | 6.8  (2.4-17.8) | NS | 3.09  (1.12-8.05) | 8.5  (2.9-21.7) |
|  | CD4^+^45RA^+^CCR7^-^ | 0.02  (0.01-0.07) | 0.05  (0.02-0.20) | 0.00004(↑) | 3.0  (0.6-6.6) | 2.6  (1.4-5.7) | 0.044  (↓) | 0.82  (0.31-3.25) | 2.0  (1.0-5.6) |
| Naïve T_c_ | CD8^+^45RA^+^CCR7^+^ | 0.13  (0.04-0.36) | 0.24  (0.06-0.66) | 0.0002(↑) | 45.2  (24.1-69.3) | 52.3  (22.5-76.8) | 0.003(↑) | 5.77  (1.63-12.46) | 47.6  (25.6-67.4) |
| Central memory | CD8^+^45RA^-^CCR7^+^ | 0.023  (0.003-0.080) | 0.030  (0.007-0.137) | 0.004(↑) | 6.6  (2.7-17.3) | 6.8 (2.0-16.5) | NS | 0.67  (0.08-3.03) | 4.9  (2.0-12.4) |
| Effectormemory | CD8^+^45RA^-^CCR7^-^ | 0.03  (0.01-0.10) | 0.06  (0.01-0.17) | 0.0002(↑) | 10.8  (5.3-26.5) | 11.8  (4.0-29.7) | NS | 1.05  (0.52-3.85) | 11.2  (4.4-26.4) |
| Effector (TEMRA) | CD8^+^45RA^+^CCR7^-^ | 0.08  (0.02-0.41) | 0.12  (0.02-0.36) | 0.036(↑) | 33.7  (13.9-53.1) | 24.4  (13.1-50.7) | 0.002 (↓) | 2.93  (0.88-14.35) | 26.0  (19.4-57.0) |
|  | CD8^+^45RO^+^CD26^++^ | 0.011  (0.001-0.088) | 0.016  (0.002-0.101) | NS | 4.1  (0.7-16.6) | 3.0  (0.7-13.3) | 0.006 (↓) | 0.24  (0.02-1.83) | 2.9  (0.3-8.3) |
| ɑβ T cells | CD3^+^TCRɑβ^+^ | 1.18  (0.58-2.14) | 2.76  (1.21-4.04) | 0.00005(↑) | 94.8  (76.6-98.9) | 96.2  (86.0-98.8) | 0.012(↑) | 52.60  (20.63-140.47) | 96.5  (86.2-98.4) |
| γδ T cells | CD3^+^4^-^8^-^TCRɣδ^+^ | 0.048  (0.004-0.118) | 0.046  (0.009-0.178) | 0.017(↑) | 3.0  (0.3-11.0) | 1.9  (0.6-6.1) | 0.0001 (↓) | 1.15  (0.30-4.20) | 2.0  (0.9-11.2) |
| Naïve T regulatorycells | CD4^+^25^+^45RA^+^FOXP3^+^ | 0.010  (0.003-0.042) | 0.019  (0.007-0.124) | 0.00008(↑) | 1.1  (0.5-4.9) | 1.0  (0.4-5.6) | NS | 0.457  (0.165-1.817) | 1.0  (0.5-6.5) |
| Effector Tregulatorycells | CD4^+^25^+^45RA^-^FOXP3^+^ | 0.030  (0.016-0.068) | 0.071  (0.027-0.178) | 0.00004(↑) | 3.8  (1.6-7.0) | 3.5  (2.2-6.9) | NS | 1.268  (0.541-4.207) | 3.4  (1.5-5.7) |
| Tr1 | CD4^+^45RA^-^49b^+^LAG3^+^ | 0.006  (0.002-0.018) | 0.011  (0.004-0.064) | 0.003(↑) | 1.4  (0.4-3.6) | 1.3  (0.4-2.9) | NS | 0.217 (<0.001-0.920) | 1.2  (0.5-3.6) |
| **B cells** |  |  |  |  |  |  |  |  |  |
| Transitional | CD19^+^24^++^38^++^ | 0.005  (0.001-0.021) | 0.013  (0.005-0.034) | 0.00004(↑) | 3.7  (1.2-10.6) | 2.6  (1.5-5.6) | 0.007 (↓) | 0.311  (0.087-1.045) | 3.0  (1.5-5.0) |
| Mature | CD19^+^24^++^38**^-^** | 0.023  (0.002-0.097) | 0.059  (0.016-0.295) | 0.00004(↑) | 14.4  (6.2-50.0) | 13.3  (4.7-35.9) | 0.0003 (↓) | 1.898  (0.319-9.011) | 13.7  (5.2-42.9) |
| Memory | CD19^+^24^+^38^+^ | 0.094  (0.022-0.274) | 0.352  (0.147-1.471) | 0.00004(↑) | 67.6  (36.6-85.7) | 76.3  (51.7-89.4) | 0.00006(↑) | 7.61  (1.52-19.09) | 74.9  (44.6-90.5) |
|  | CD19^+^27^+^ | 0.027  (0.003-0.131) | 0.067  (0.018-0.459) | 0.00004(↑) | 20.4  (5.5-63.1) | 14.4  (3.9-54.8) | 0.00004 (↓) | 1.055  (0.535-13.742) | 13.0  (3.5-52.0) |
| IL-2R^+^ | CD19^+^25^+^ | 0.002  (<0.001-0.017) | 0.002  (0.001-0.043) | NS | 1.4  (0.4-7.5) | 0.6  (0.1-5.0) | 0.0004 (↓) | 0.065  (0.012-1.028) | 0.6  (0.1-3.9) |
| IL-2R^w^ | CD19^+^25^w^ | 0.017  (0.002-0.070) | 0.031  (0.007-0.228) | 0.0001(↑) | 10.3  (5.5-33.8) | 7.4  (2.9-27.2) | 0.00005 (↓) | 0.671  (0.196-6.422) | 6.8  (3.0-24.3) |
| **NK cells** |  |  |  |  |  |  |  |  |  |
| Cytolytic | CD56^+^16^++^ | 0.191  (0.025-0.447) | 0.201  (0.025-0.521) | NS | 38.9  (5.0-73.3) | 17.9  (2.0-53.5) | 0.00008 (↓) | 3.901  (0.882-11.986) | 12.5  (3.2-52.9) |
| Cytokineproducing | CD56^++^16^+^ | 0.018  (0.006-0.038) | 0.029  (0.005-0.230) | 0.001(↑) | 3.4  (1.3-13.9) | 2.3  (0.3-12.7) | NS | 0.619  (0.261-2.117) | 2.2  (0.5-7.2) |
| Invariant NKT (iNKT) | CD3^+^Vα24^+.^ | 0.003  (0.001-0.022) | 0.003  (0.001-0.023) | NS | 0.26  (0.05-1.28) | 0.16  (0.02-1.03) | 0.016 (↓) | 0.089  (0.006-2.147) | 0.15  (0.02-6.86) |

(↑) = significant increased concentration, (↓) = significant decreased concentration, NS = non-significant

**Supplementary Table 5:** The effect of stem cell mobilization with G-CSF on the intracellular cytokine concentrations in lymphoid cells from healthy donors (n = 22). From left to right, the results for T helper cells (T_H_), T cytotoxic cells (T_C_), CD3^+^4^-^8^-^ T cells, B cells and CD3^-^19^-^ cells are presented. For each cytokine the median untreated concentrations (x 10^9^/L) of positive cells are shown together with the concentrations during G-CSF treatment. On the line below the same values are presented as median percentages of their parent populations acquired by flow cytometry.The Wilcoxon's test for paired samples was used for comparison of pre-treatment and G-CSF treated/pre-apheresis concentrations and percentages.

|  | **T_H_ cells** | | | **T_c_ cells** | | | **CD3^+^4^-^8^-^ T cells** | | | **B cells** | | | **CD3^-^19^-^cells** | | |
| --- | --- | --- | --- | --- | --- | --- | --- | --- | --- | --- | --- | --- | --- | --- | --- |
| **Cytokine** | **Prior to G-CSF** | **During G-CSF** | **P** | **Prior to G-CSF** | **During G-CSF** | **P** | **Prior to G-CSF** | **During**  **G-CSF** | **P** | **Prior to G-CSF** | **During G-CSF** | **P** | **Prior to G-CSF** | **During G-CSF** | **P** |
| IFNɣ | 0.157/  20.6 | 0.374/  20.6 | 0.00004 (↑)/  NS | 0.154/  60.4 | 0.302/  56.2 | 0.001 (↑)/  0.018 (↓) | 0.030/  69.2 | 0.040/  61.3 | 0.022 (↑)/  0.001(↓) | 0.0013/  1.9 | 0.0036/  1.1 | 0.002 (↑)/  0.025(↓) | 0.0789/  41.8 | 0.0776/  32.1 | NS/  0.002 (↓) |
| IL10 | 0.0042/  0.5 | 0.0100/  0.7 | 0.00004 (↑)/  0.006 (↑) | 0.0010/  0.64 | 0.0019/  0.8 | 0.000061 (↑)/  0.029 (↑) | 0.0001/  0.4 | 0.0003/  0.4 | 0.001 (↑)/  NS | 0.0012/  6.7 | 0.0041/  4.4 | 0.001 (↑)/  NS | 0.0005/  0.3 | 0.0010/  0.4 | 0.005 (↑)/  0.004 (↑) |
| TGFβ | 0.178/  24.1 | 0.367/  24.2 | 0.000061 (↑)/  NS | 0.112/  43.94 | 0.231/  40.3 | 0.004 (↑)/  NS | 0.0150/  50.9 | 0.0297/  48.9 | 0.024 (↑)/  NS | 0.0130/  10.5 | 0.0249/  6.5 | 0.000295 (↑)/  0.001 (↓) | 0.0043/  3.9 | 0.0033/  2.4 | 0.022 (↑)/  0.003 (↓) |
| IL4 | 0.0175/  2.7 | 0.0322/  2.0 | 0.000367 (↑)/ NS | 0.0053/  2.8 | 0.0070/  2.2 | NS/  0.039 (↓) | 0.0002/  1.0 | 0.0002/  0.8 | NS/  NS | 0.0013/  2.0 | 0.0049/  1.8 | 0.000069 (↑)  NS | 0.0039/  2.9 | 0.0078/  4.6 | 0.003 (↑)/  0.030 (↑) |
| IL9 | 0.0048/  1.1 | 0.0152/  1.1 | 0.001 (↑)/ 0.024 (↑) | 0.0112/  4.8 | 0.0107/  4.3 | 0.011 (↓) /  NS | 0.0025/  16.6 | 0.0055/  14.3 | NS/  NS | 0.0011/  1.0 | 0.0012/  0.4 | NS/  0.001(↓) | 0.0925/  50.0 | 0.1086/  40.3 | NS/  0.011 (↓) |
| IL17 | 0.0091/  1.2 | 0.0269/  1.5 | 0.000046 (↑)/  NS | 0.0008/  0.3 | 0.0014/  0.5 | 0.005 (↑)/  NS | 0.0001/  0.5 | 0.0004/  0.8 | 0.007 (↑)/  0.016 (↑) | 0.0001/  0.1 | 0.0003/  0.1 | 0.001 (↑)/  NS | 0.0001/  0.04 | 0.0004/  0.04 | NS/  NS |
| IL22 | 0.0153/  2.6 | 0.0211/  1.6 | NS/  NS | 0.0033/  1.1 | 0.0034/  0.9 | NS/  0.050 (↓) | 0.0001/  0.4 | 0.0001/  0.3 | NS/  NS | 0.0010/  1.1 | 0.0030/  0.9 | 0.000187 (↑)/  NS | 0.0006/  0.4 | 0.0006/  0.4 | NS/  NS |

(↑) = significant increased concentration, (↓) = significant decreased concentration, NS = non-significant
