# Supplementary figures and images for: Peripheral Blood Stem Cell Mobilization in Healthy Donors by Granulocyte Colony-Stimulating Factor Causes Preferential Mobilization of Lymphocyte Subsets
Source: Front Immunol. 2018 May 2;9:845. doi: 10.3389/fimmu.2018.00845 (PMC5941969; doi:10.3389/fimmu.2018.00845)

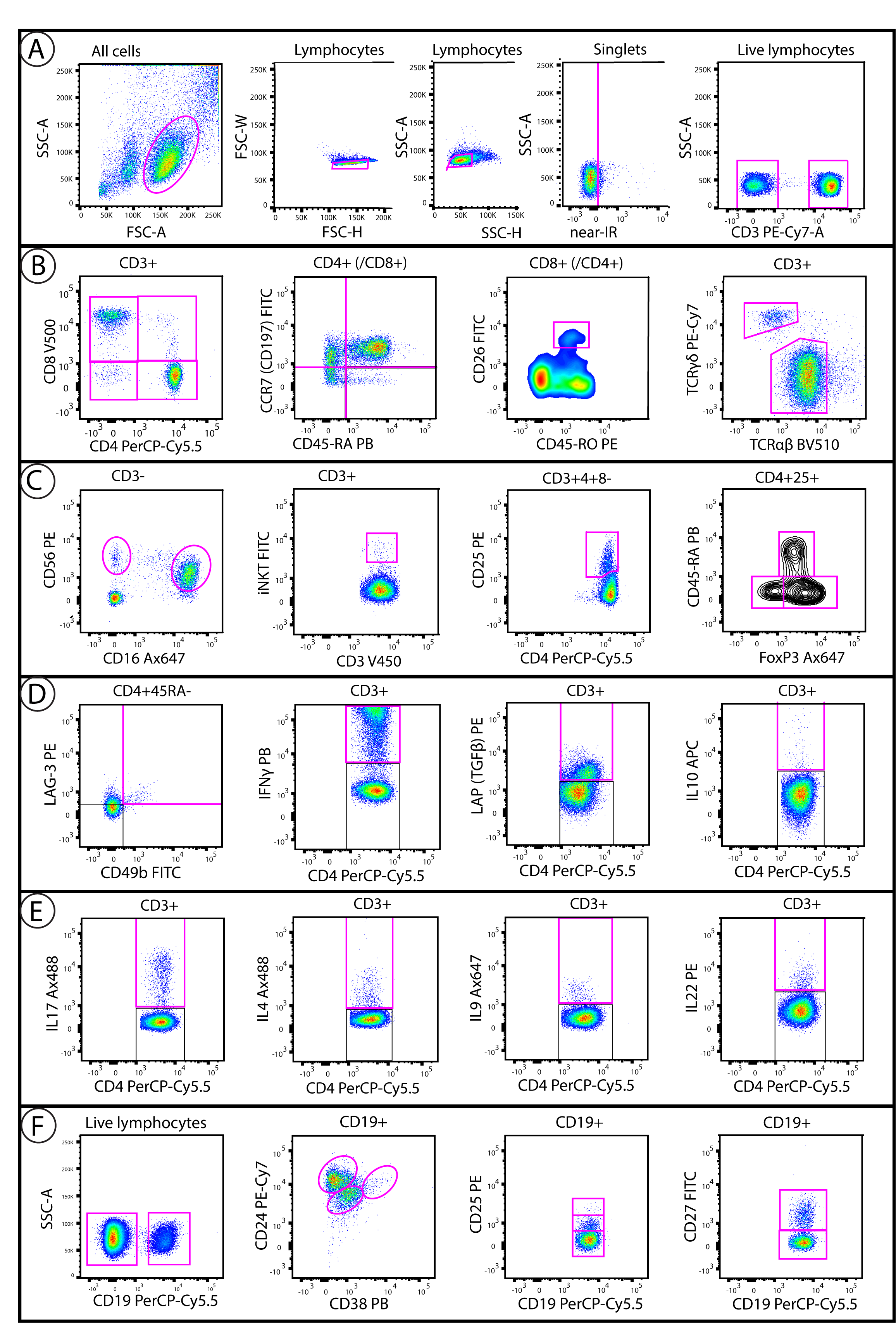

Supplement: Supplementary file 1 [file Data_Sheet_1.zip › Supplementary Figure 1.TIF]

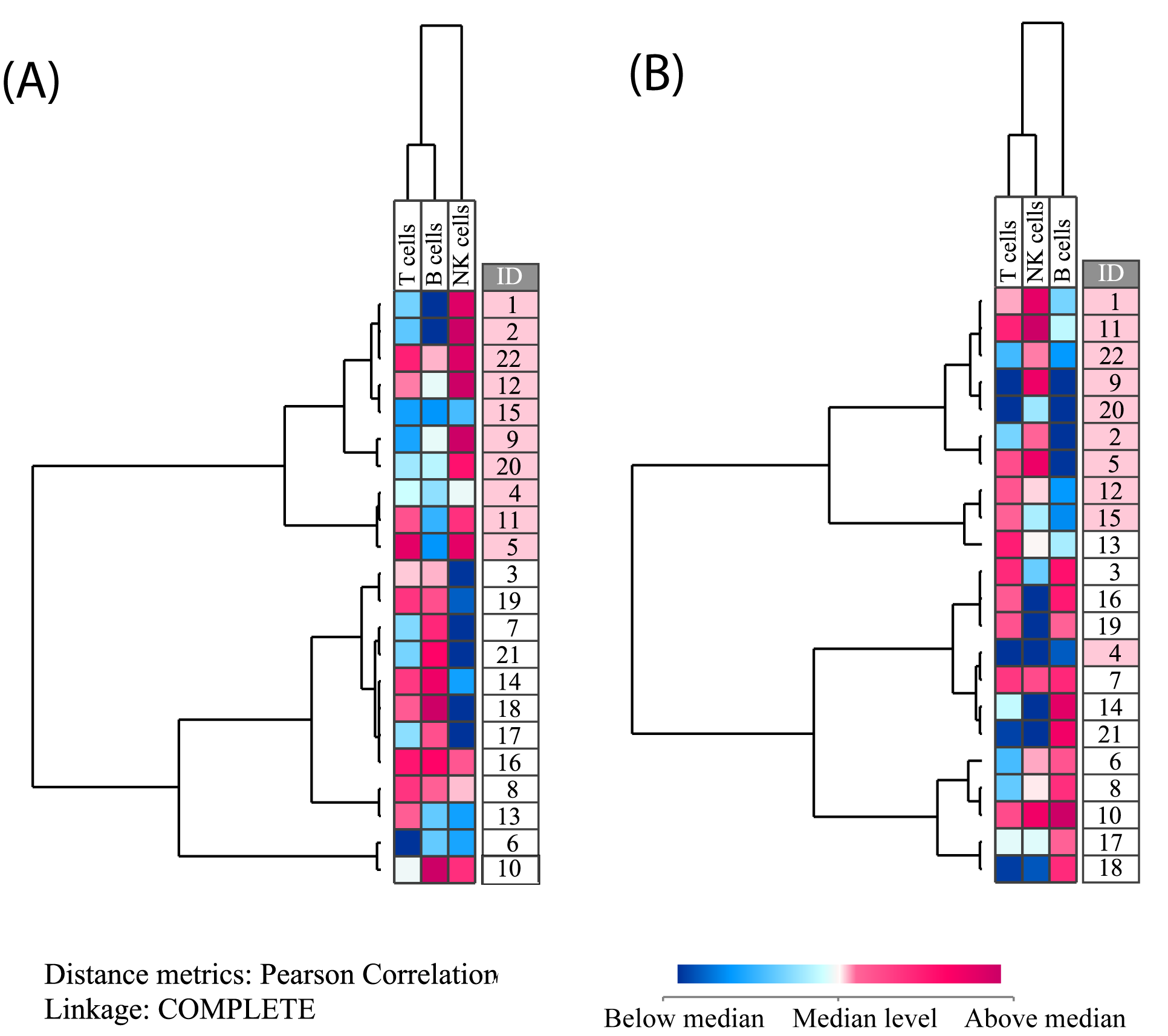

Supplement: Supplementary file 1 [file Data_Sheet_1.zip › Supplementary Figure 2.TIF]
